# Prognostic Significance of Neutrophil-to-Lymphocyte Ratio in Colorectal Liver Metastasis: A Systematic Review and Meta-Analysis

**DOI:** 10.1371/journal.pone.0159447

**Published:** 2016-07-18

**Authors:** Haowen Tang, Bingmin Li, Aiqun Zhang, Wenping Lu, Canhong Xiang, Jiahong Dong

**Affiliations:** 1 Hospital and Institute of Hepatobiliary Surgery, Chinese PLA General Hospital, Chinese PLA Medical School, 28 Fuxing Road, Haidian, Beijing, 100853, China; 2 Chinese PLA Medical School, 28 Fuxing Road, Haidian, Beijing, 100853, China; 3 Center for Hepatopancreatobiliary Diseases, Beijing Tsinghua Changgung Hospital, Tsinghua University Medical Center, 168 Litang Road, Changping, Beijing, 102218, China; The University of Hong Kong, HONG KONG

## Abstract

**Background and Objective:**

Inflammation is deemed to play critical roles in tumor progression and metastasis, and an increased neutrophil-lymphocyte ratio (NLR) has been reported to correlate with poor survivals in various malignancies. However, association between NLR elevation and survival outcome in patients with colorectal liver metastasis (CRLM) remains controversial. The aim of this study was to investigate the prognostic significance of elevated NLR in CRLM.

**Methods:**

The meta-analysis was conducted in adherence to the MOOSE guidelines. PubMed, Embase, Cochrane Library, Web of Science and the Chinese SinoMed were systematically searched to identify eligible studies from the initiation of the databases to May, 2016. Overall survival (OS) and recurrence free survival (RFS) were pooled by using hazard ratio (HR) with corresponding 95% confidence interval (CI). Correlation between NLR values and clinicopathological features was synthesized by using odds ratio (OR) with corresponding 95% CI.

**Results:**

A total of 1685 patients from 8 studies (9 cohorts) were analyzed, consisting 347 (20.59%) in high pretreatment NLR value group and 1338 (79.41%) in low pretreatment NLR value one. The results demonstrated that elevated pretreatment NLR was significantly related to poor OS (HR 2.17, 95% CI 1.82–2.58) and RFS (HR 1.96, 95% CI 1.64–2.35) in patients with CRLM.

**Conclusion:**

The result of this systematic review and meta-analysis indicated that an elevated pretreatment NLR was closely correlated with poor long-term survival (OS and RFS) in CRLM patients. NLR can be routinely monitored and serve as a useful and cost-effective marker with strong prognostic significance in patients with CRLM.

## Introduction

Colorectal cancer (CRC) is one of the most common human malignancies. Worldwide, approximately 1.2 million new cases are diagnosed and over 600 thousand deaths are estimated to occur annually [1]. About 40% patients with CRC develop liver metastasis at the time of presentation, with approximately 20% presenting as synchronous metastasis (within 6 months of resection of the primary tumor) and the remaining 20% as metachronous metastasis (after this period) [2–4]. Despite of therapeutic advances and multidisciplinary collaborations, the prognosis of colorectal liver metastasis (CRLM) still remains unsatisfactory, with a 5-year survival rate of 25–47% and a median survival of 33–49.8 months after resection of liver metastases [5–9]. Furthermore, recurrences occur in over onehalf of patients (56.7%) after resection of liver metastases within 2 years [10]. Therefore, identification of a reliable prognostic predictor for CRLM to help clinicians implement preventive and therapeutic measures for risk patients is of considerable importance and urgent need.

Inflammatory response is deemed to play critical roles in tumor progression and metastasis [11,12] and that systemic inflammation has been proven to be in relationship with poor survivals in various tumors [13–16]. Increased levels of pro-inflammatory cytokines and signaling molecules in cancer patients on one hand might reflect both disease activity and the innate response of the host to the tumor [17], and on the other hand could promote neoangiogenesis or lymphangiogenesis [11,18]. Recent published literature has documented a correlation between pretreatment systemic inflammation and worse cancer-specific survival in patients with CRLM; furthermore, the authors concluded that patients with higher tumor burden (lager tumor number and/or size) were more likely to express systemic inflammatory responses to tumor, which reflected the aggressiveness of tumor and influenced the prognosis [19]. Hence, systemic inflammation markers could serve as promising prognostic predictors of patients with CRLM. Neutrophil-lymphocyte ratio (NLR) reflecting general immune response to various stress stimuli has been proven to be a prognosis-related marker, its elevation correlating with poor survivals in various malignancies (hepatocellular carcinoma, cholangiocarcinoma, pancreatic cancer, esophageal cancer and colorectal cancer) [20–25]. However, controversy still exists on influence of elevated NLR on long-term outcome in CRLM. Previous attempts to investigate the prognostic significance of NLR have yielded conflicting results. Earlier reports described that heightened NLR remained an independent predictor of poor OS for patients with CRLM [26,27], whereas Neofytou K and colleagues failed to demonstrate such predictive value [28]. Given this, a meta-analysis was conducted to reveal the prognostic significance of NLR for CRLM. Additionally, correlations between NLR values and clinicopathological features were assessed.

## Methods

The meta-analysis was conducted in adherence to the recommendations of the Meta-analysis of Observational Studies in Epidemiology group (MOOSE) guidelines [29]. To ensure accuracy and minimize bias, all vital stages of the analysis were carried out separately by two reviewers; any disagreement was settled through consensus discussion.

### Study Identification

A systematic literature search of PubMed, EMBASE, the Cochrane Library, Web of Science and the Chinese SinoMed was performed to select relevant articles from the initiation of the databases to May, 2016. No additional restrictions were applied to the searches with regard to region, publication type or language. The following medical subject headings (MeSH) or keywords were used: ‘‘Colorectal neoplasms,” “Neoplasm metastasis,” “Liver neoplasm,” “Colorectal liver metastasis,” “Liver metastasis from/of colorect*,” “Liver metastasis from/of colon/rectum,” “CRLM,” “CLM,” “Neutrophil to Lymphocyte Ratio,” “Neutrophil-lymphocyte Ratio,” “Neutrophil Lymphocyte Ratio,” and “NLR.” In addition, the references given in the retrieved papers were manually checked for further relevant articles. In the case of repeated studies describing the same population, only the most recent or the highest in quality was included. The latest search was performed on May 19, 2016. To ensure the reliability and verifiability of our analysis, eligible studies were identified in accordance with the following inclusion and exclusion criteria. The inclusion criteria were: (1) patients with a pathologically confirmed diagnosis of CRLM. (2) pretreatment NLR provided and included as a variable in outcome analysis. (3) cutoff values of NLR clearly reported. (4) hazard ratio (HR) values describing association between pretreatment NLR and survival outcomes (overall survival (OS) and/or recurrence free survival (RFS)) available or obtainable from other information presented. A study must meet all four inclusion criteria for inclusion. The exclusion criteria were: (1) nonhuman experiments. (2) review articles, letters, case reports, editorials or comments and conference abstracts and studies irrelevant to our topic. (3) overlapping or duplicate reports. (4) articles including patients mainly undergoing repeated hepatectomy for CRLM or with extrahepatic metastasis. (5) known active infections or other inflammatory conditions were diagnosed at the time of blood sampling for NLR. A study meeting any of the five exclusion criteria was excluded.

### Data Extraction and Definition

The following relevant parameters were extracted and summarized independently by two reviewers (HW T and BM L) for each study included in the meta-analysis: first author, year of publication, type of study, study region, recruitment period, predominant treatment method, total patients, mean or median age, proportion of male patients, sampling time and site, cutoff value, number of patients with elevated NLR, surveillance endpoint, HR and corresponding 95% confidence interval (CI) values, and follow-up length. Outcomes from multivariate analyses were superior to those from univariate analyses for inclusion if both were presented. If HR, 95% CI, or additional key data were absent from an article, the corresponding author of the report was contacted by e-mail. In the absence of replies from the authors, the methods introduced by Tierney were used to derive an approximate estimation of the HR and corresponding 95% CI from other information, such as the OS Kaplan-Meier curve [30].

In the present study, NLR was defined as the serum absolute neutrophil count divided by the serum absolute lymphocyte count in peripheral blood [31]. OS was defined as the interval between the medical treatments (surgical resection, chemotherapy or percutaneous radiofrequency ablation (RFA)) and the death or the last observation of patients. RFS was calculated from the date of curative treatment until the detection of tumor recurrence. Quality assessment for each article included was conducted using the Newcastle-Ottawa Scale (NOS) that was mainly concerned with three aspects (selection of patients, comparability of groups, and assessment of outcomes). Studies scored with six or more were considered to be of high quality. Subgroups were generated if at least two studies (or two cohorts) were available; otherwise, subgroups analyses were not performed. A two-tailed P value less than 0.05 was considered statistically significant.

### Outcome Comparison and Statistical Analysis

For the comparison of OS and RFS, the HR with corresponding 95% CI was used. An HR value (reference: low pretreatment NLR value group) greater than 1 indicated a strong association between high NLR value and poor survival outcome. For the analysis of the correlation between NLR values and clinicopathological features, odds ratio (OR) with corresponding 95% CI was synthesized as the effective value. STATA statistical software (version 12.0, Stata Corporation, College Station, TX, USA) was utilized to conduct the meta-analysis. Between-study heterogeneity was explored by Cochrane’s Q and I^2^ tests. A fixed effect model was used in the absence of significant heterogeneity (I^2^ <50%); otherwise, a random effect model was used. Begg’s funnel plot and Egger’s test were used to assess publication bias. Sensitivity analysis was performed by omitting studies included one by one. Subgroup analyses were performed to explore the between-study heterogeneity according to predefined parameters: predominant treatment method (surgical resection and non-surgical treatment), sample size (size ≥100 and size <100), NLR cutoff value (value = 5 and value = 2.5). Meta-regression analysis was not conducted due to the limited number of studies; this analysis is best suited to analyzing at least 10 studies.

## Results

### Study Selection and Patients Characteristics

**Fig 1:**
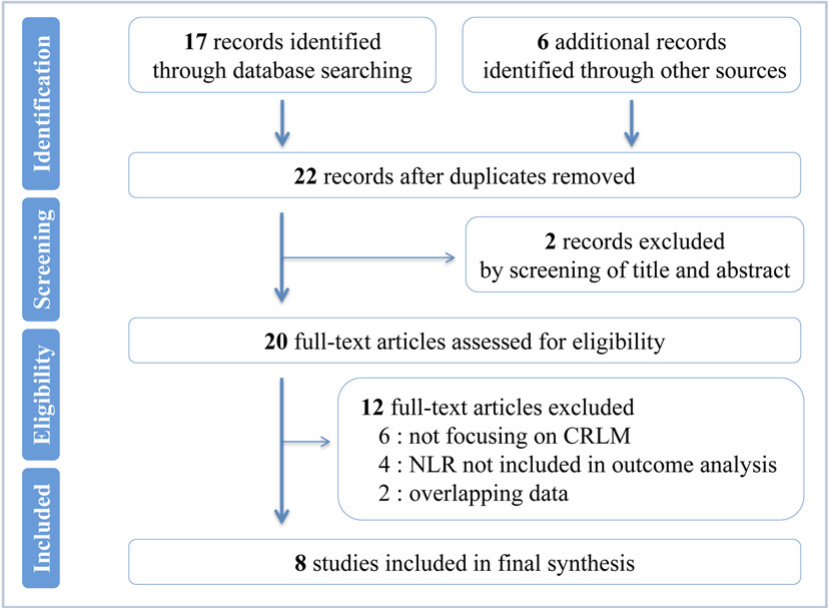
Flowchart of the study selection. Systematic search and selection of relevant articles.

A flowchart of the study selection is shown in Fig 1. The search returned a total of 22 references. By meticulously screening the titles and abstracts, two references were eliminated. Among the remaining 20 potentially appropriate studies, 12 were excluded by full-text analysis because they matched one of the exclusion criteria. Finally, eight studies reporting on 1685 patients were eligible to be included in the present meta-analysis [26,27,32–37]. Among the total eight studies published between 2008 and 2015, one was prospective nonrandomized studies and seven were retrospective; 11 were English-language literatures and one was Chinese-language (from the database of the Chinese SinoMed) [33]. The studies were conducted in the UK (4 studies), Poland (1 study), the USA (1 study) and China (2 studies). As the study by Kishi Y [37] included two cohorts (surgical resection cohort and chemotherapy cohort) and documented the HR and 95% CI individually, we tagged them as Kishi Y (SR) and Kishi Y (Non SR), respectively. And two studies by Neal CP reporting on different groups of patients from different recruitment periods were marked as Neal CP (2015) [32] and Neal CP (2011) [36], respectively. Hence, a total of 1685 patients from eight studies (nine cohorts) were analyzed, consisting 347 (20.59%) in high pretreatment NLR value group and 1338 (79.41%) in low pretreatment NLR value one. The median of sample size for these studies is 169 (range 90–440). For most studies, the mean or median age of the patients was in the 50s or 60s, and the median of male patient percentage was 63.58% (range 57.14%-67.78%). Of all the patients analyzed, 1405 in six cohorts underwent surgical resection and 280 in three cohorts received chemotherapy or percutaneous RFA. In the majority of the studies included, the patients received the treatments (surgical and non-surgical) for the CRLM after curative colorectal resection of primary tumors. The relationship between NLR and OS was explored in seven cohorts of 1491 patients, and six cohorts reporting on 1093 patients analyzed the association between NLR and RFS. The median follow-up length of the studies was 29.7 months (range 16–44). NLR values were all evaluated prior to therapy; the cutoff values were defined by different methods in each study. Study characteristics, patient demographic information and quality assessment were summarized in S1 Table; main outcomes were outlined in Table 1.

**Table 1:** Results of the meta-analysis on prognostic significance of NLR in CRLM.

	**Overall Survival**						**Recurrence Free Survival**					
	**No. studies**	**No. patients**	**HR**	**95% CI**	I^2^,%	P value for heterogeneity	**No. studies**	**No. patients**	**HR**	**95% CI**	I^2^,%	P value for heterogeneity
**Overall**	7	1491	2.17	1.82–2.58	0	0.66	6	1093	1.96	1.64–2.35	45.20	0.10
**Treatment methods**												
Surgical resection	5	1309	2.08	1.73–2.49	0	0.74	4	903	1.89	1.34–2.66	58.60	0.07
Non-surgical treatment	2	182	3.11	1.83–5.29	0	0.71	2	190	2.33	1.37–3.95	29.90	0.23
**Sample size**												
≥100	5	1309	2.08	1.73–2.49	0	0.74	3	286	1.79	1.24–2.59	68.20	0.04
<100	2	182	3.11	1.83–5.29	0	0.71	3	807	2.48	1.55–3.98	0	0.42
**NLR cutoff**												
= 5	6	1322	2.17	1.81–2.60	0	0.53	4	826	2.27	1.84–2.80	0	0.69
= 2.5	1	-	-	-	-	-	2	267	1.30	0.91–1.85	0	0.46

NLR: neutrophil-lymphocyte ratio; HR: hazard ratio; CI: confidence interval.

### NLR and OS

**Fig 2:**
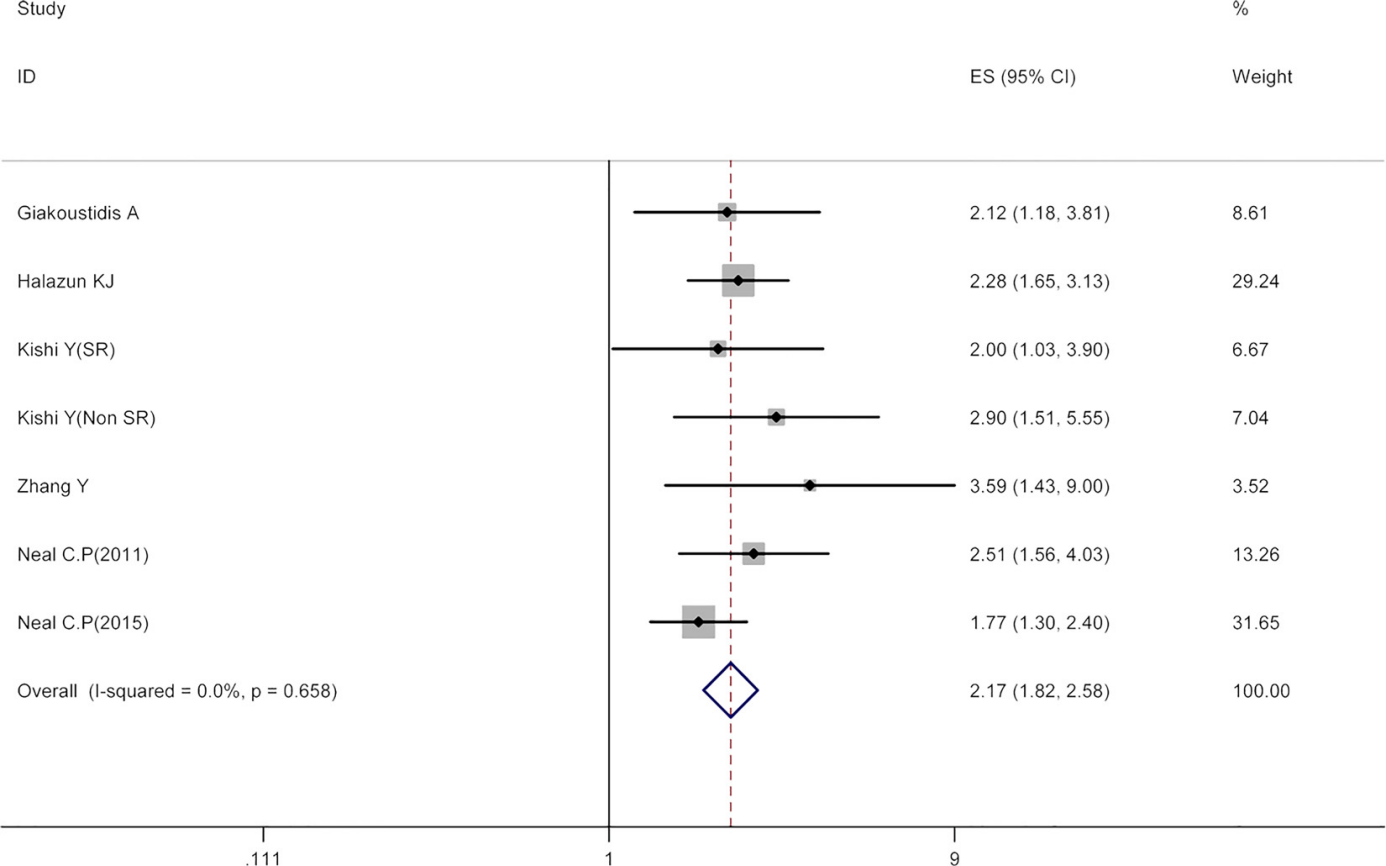
Results of the meta-analysis on pooled HR values for OS. Each square denotes the HR for that trial comparison with the horizontal lines showing the 95% CIs. The size of the square is directly proportional to the amount of information contributed by the trial. The blue hollow diamond gives the pooled HR from the fixed effect model; the centre of this diamond denotes the HR and the extremities the 95% CI.

Seven cohorts covering 1491 patients described the association between NLR and OS in CRLM. The pooled HR for high pretreatment NLR value group was found to be 2.17 (95% CI 1.82–2.58) when compared with low pretreatment NLR value group with no between-study heterogeneity (I^2^ 0, P = 0.66). The result indicated that a high pretreatment NLR value was significantly related to inferior OS in patients with CRLM. Fig 2 showed the results of the pooled analysis.

### NLR and RFS

**Fig 3:**
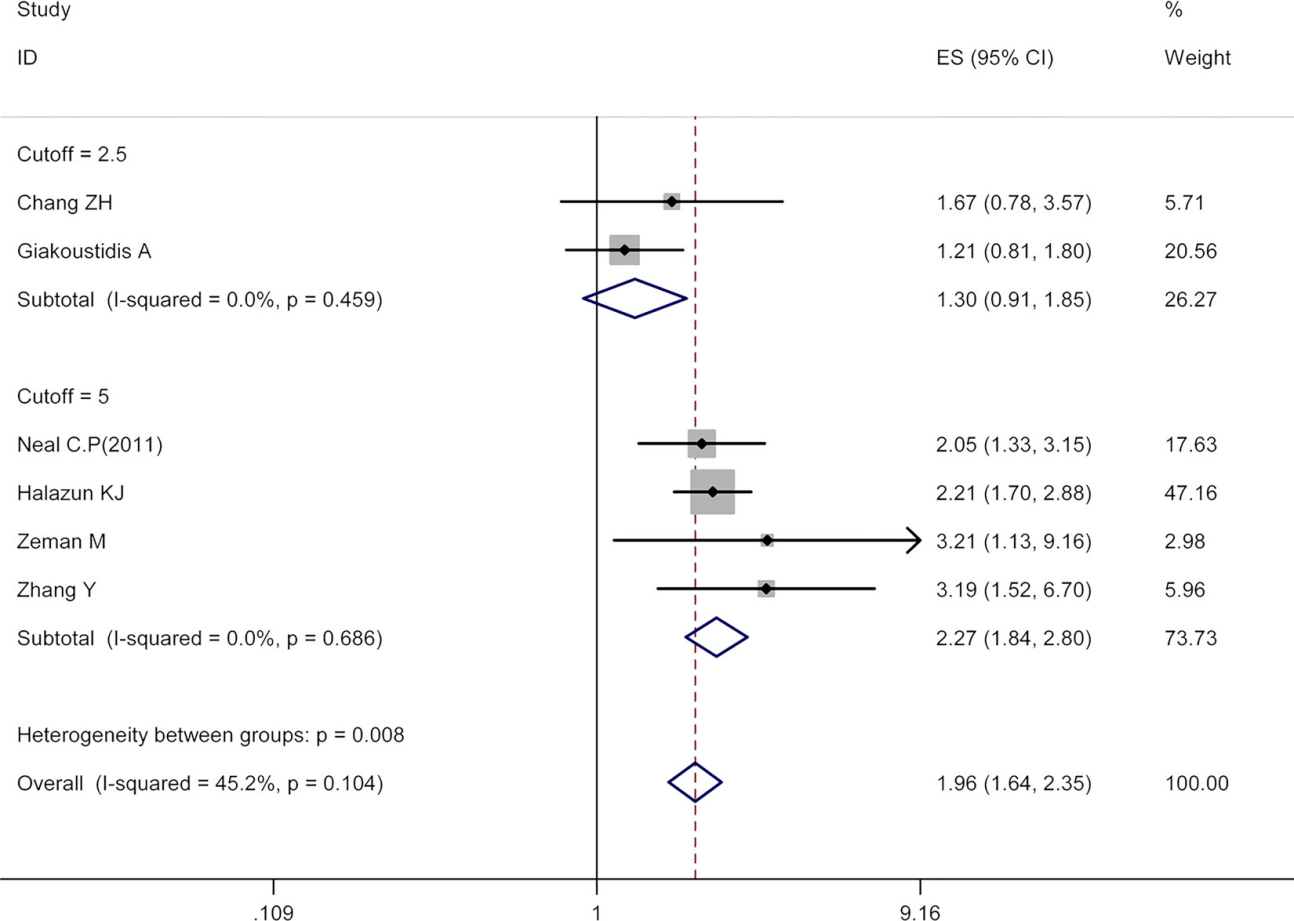
Results of the meta-analysis on pooled HR values for RFS. Results of overall analysis and subgroup analysis (cutoff value = 2.5 and cutoff value = 5). Each square denotes the HR for that trial comparison with the horizontal lines showing the 95% CIs. The size of the square is directly proportional to the amount of information contributed by the trial. The bottom blue hollow diamond gives the pooled HR from the fixed effect model; the centre of this diamond denotes the HR and the extremities the 95% CI.

The relationship between NLR and RFS was explored in six cohorts (1093 patients). As illustrated in Fig 3, the pooled estimate for high pretreatment NLR value group was of statistical significance (HR 1.96, 95% CI 1.64–2.35) with moderate between-study heterogeneity (I^2^ 45.20%, P = 0.10), indicating that patients with high pretreatment NLR were associated with worse RFS.

### Subgroup Analyses

In accordance with three predefined parameters: predominant treatment method (surgical resection and non-surgical treatment), sample size (size≥100 and size<100), NLR cutoff value (value = 5 and value = 2.5), subgroup analyses were conducted to investigate the associations between NLR and prognosis. With respect to OS, five subgroup analyses (subgroup analysis of NLR cutoff value = 2.5 was cancelled due to only one cohort for inclusion) were conducted, their results uniformly revealing survival superiority in patients with low pretreatment NLR value. Regarding RFS, pooled analyses showed similar results in comparison with those for OS except that no significant differences in RFS were identified between high NLR value group and low value one in the subgroup of NLR cutoff value = 2.5 (HR 1.30, 95% CI 0.91–1.85). All the above results were detailed in Table 1.

### Correlation between NLR Values and Clinicopathological Features

**Fig 4:**
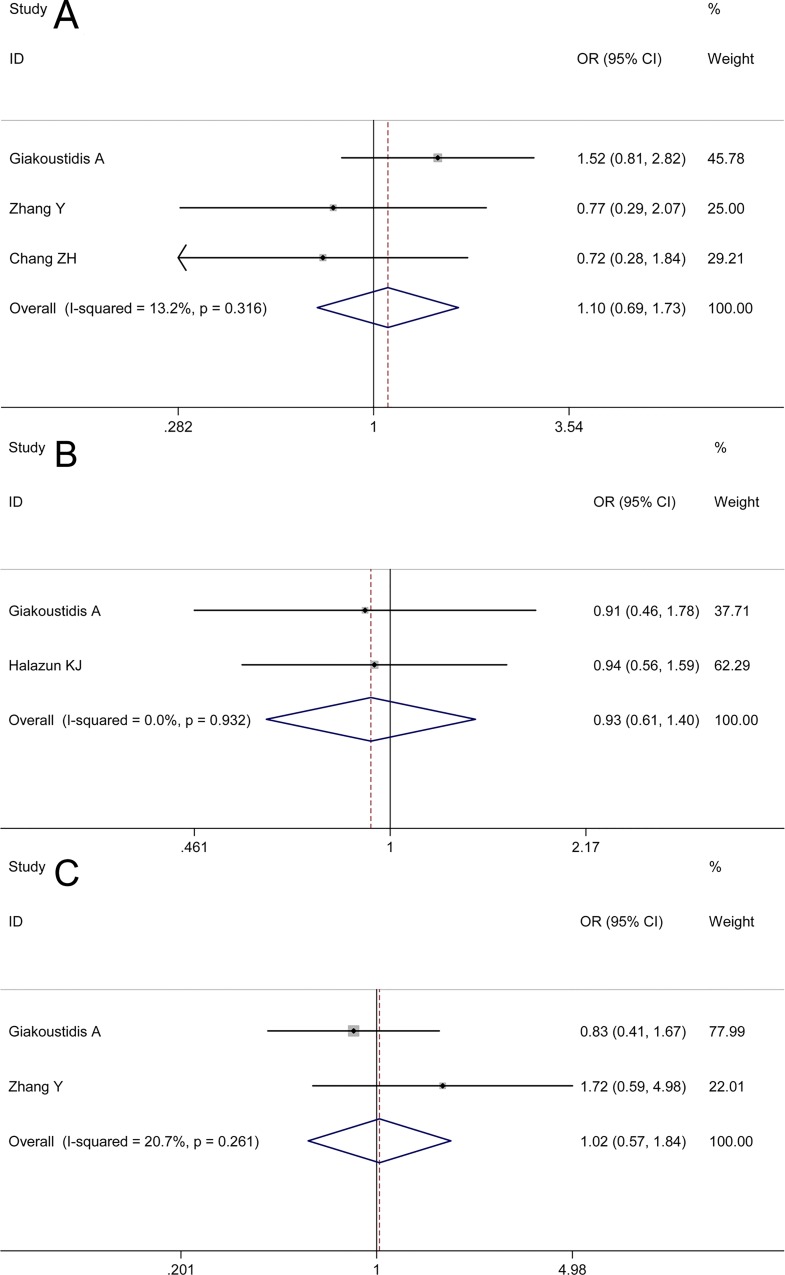
Correlation between NLR values and clinicopathological features. Fig 4A denotes the relationship between NLR value and pretreatment carcinoembryonic antigen (CEA) level (≤5 ng/mL and >5 ng/mL), the result of which showed that associations between high NLR values and higher CEA levels were with marginal significance. Fig 4B denotes the relationship between NLR value and timing of CRC metastasis (synchronous type and metachronous type), the result of which demonstrates no tendency of heightened pretreatment NLR towards either of the two types. Fig 4C denotes the relationship between NLR value and number of metastasis at diagnosis (metastases ≥3 and metastases <3), the result of which showed no significant correlation between elevated pretreatment NLR and number of metastasis.

Data with reference to the relationship between NLR value and pretreatment carcinoembryonic antigen (CEA) level (≤5 ng/mL and >5 ng/mL) were reported in three studies. The analysis returned an OR of 1.10 in favor of high NLR value group with a slight between-study heterogeneity (I^2^ 13.20%, P = 0.32); however, the 95% CI crossed the no-effect line (Fig 4A, 95% CI 0.69–1.73), which showed that associations between high NLR values and higher CEA levels were with marginal significance.

Two studies were pooled to find a combined effect on the relationship between NLR value and timing of CRC metastasis (synchronous type and metachronous type). The pooled result did not demonstrate a tendency of heightened pretreatment NLR towards either of the two types (Fig 4B, OR 0.93, 95% CI 0.61–1.40) with no between-study heterogeneity (I^2^ 0.00%, P = 0.93).

With regards to number of metastasis at diagnosis (metastases≥3 and metastases<3), data from two studies were put into the overall analysis. A similar result showing no significant correlation between elevated pretreatment NLR and number of metastasis was also produced (Fig 4C, OR 1.02, 95% CI 0.57–1.84) with no obvious between-study heterogeneity (I^2^ 20.70%, P = 0.26).

### Analysis of Sensitivity and Test for Publication Bias

**Fig 5:**
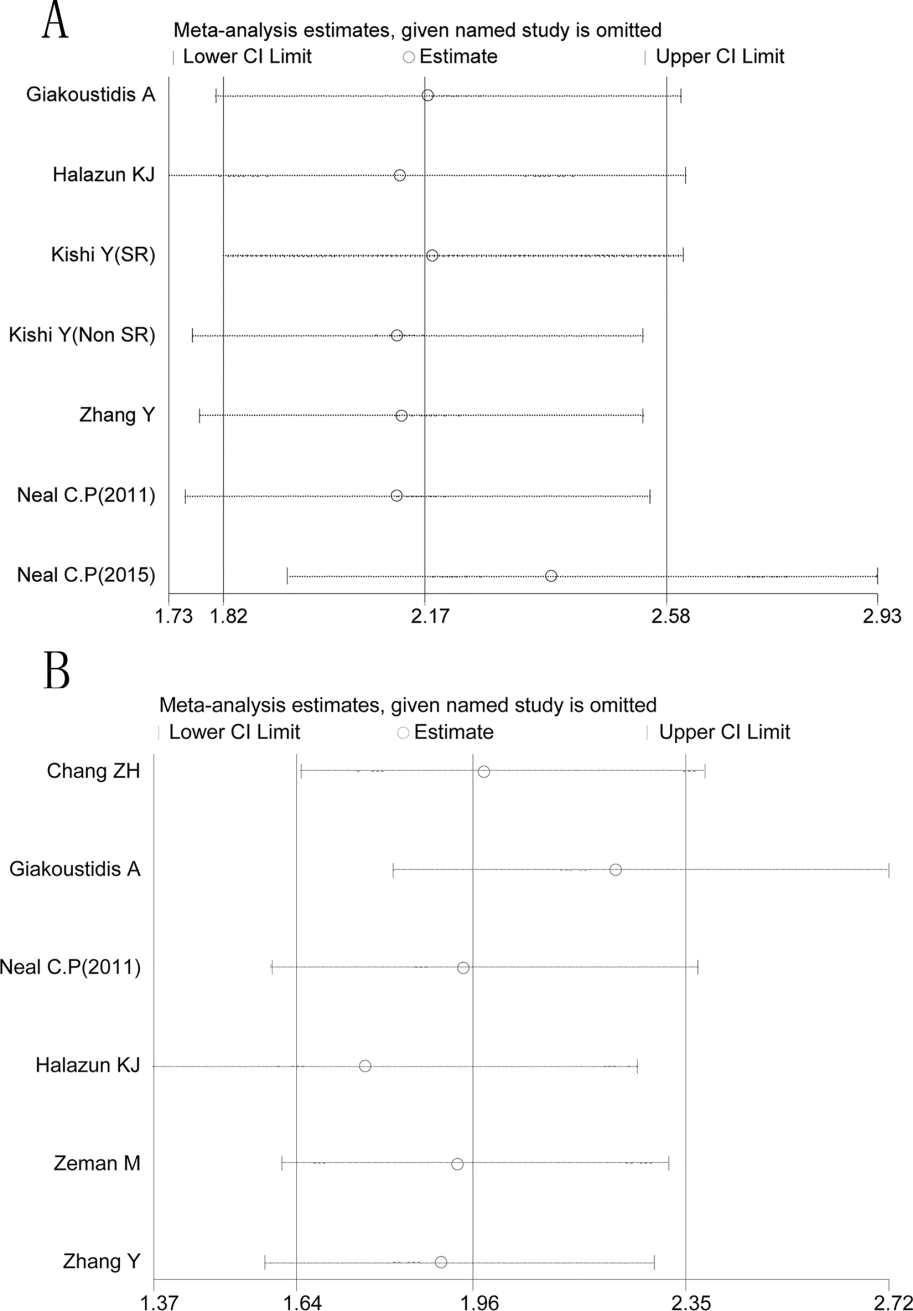
Result of sensitivity analysis. Fig 5A denotes the results of OS in sensitivity analysis; Fig 5B denotes the results of RFS in sensitivity analysis. The middle vertical line indicates the combined HR, and the two vertical lines represent the corresponding 95% CI values. The middle small circle and two ends of the dotted lines indicates the pooled HR and 95% CI values, respectively, when the study on the left was omitted after each round of analysis.

**Fig 6:**
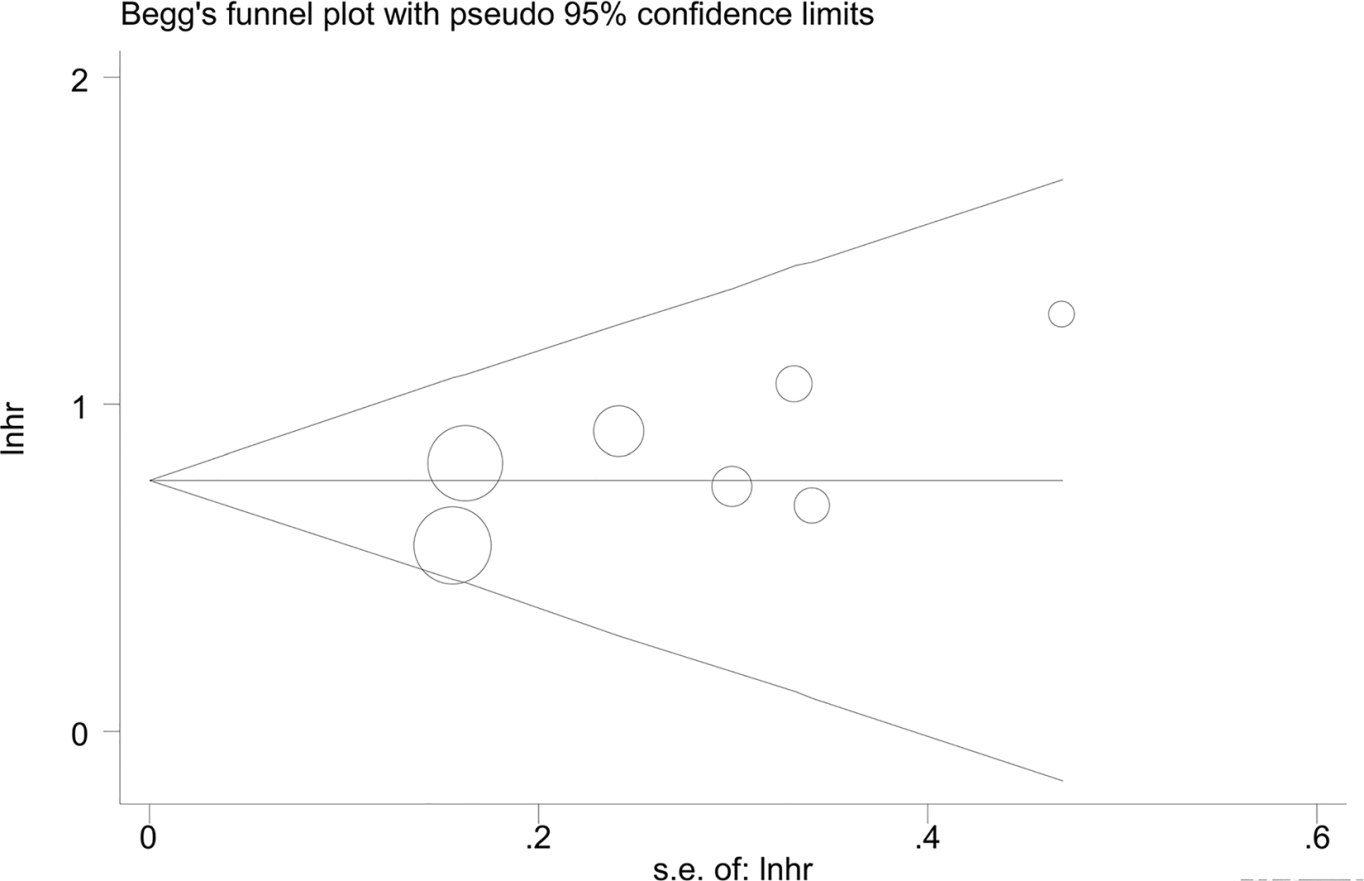
Begg's funnel plot to evaluate OS. Funnel plot showing symmetry indicative of no evidence of publication bias for OS.

By omitting the included studies sequentially, a sensitivity analysis aiming to evaluate the impact of a single study on the overall pooled HR was performed. No significant changes of HR values were produced by exclusion of any single study, with a range from 2.12 to 2.38 of HRs in OS (Fig 5A) and a range from 1.76 to 2.22 of HRs in RFS (Fig 5B). There was no evident publication bias by Eegg’s test (OS: P = 0.12, RFS: P = 0.75), with symmetry in Begg’s funnel plot, as shown in Fig 6.

## Discussion

The predictive value of NLR has been identified in various tumors recently. However, prognostic significance of NLR in CRLM still remains unclear. To the best of our knowledge, this is the first meta-analysis investigating the association between NLR and survival outcome as well as clinicopathological features in CRLM patients. The results demonstrated that increased pretreatment NLR was closely linked with worse long-term survival (OS and RFS) in patients with CRLM. The between-study heterogeneities of the pooled analysis were not obvious (OS: I^2^ 0, RFS: I^2^ 45.20%), which was confirmed by the results of subgroup analyses. Most of the results remained unaffected in the subgroup analyses based on predominant treatment method (surgical resection and non-surgical treatment), sample size (size≥100 and size<100), NLR cutoff value (value = 5), and a commensurate results were produced by sensitivity analysis. However, no significant correlations between NLR and the pretreatment CEA level, the timing of CRC metastasis and the number of metastasis at diagnosis were detected. Taken together, the present study showed that patients with high pretreatment NLR carried a long-term survival inferiority when compared to those with low pretreatment NLR (HR 1.49, 95% CI 1.36–1.63), indicating that NLR might be a promising prognostic marker in patients with CRLM.

The potential definite reasons for the correlation between NLR elevation and inferior survival outcome in patients with CRLM are complex and have not been well clarified. The following possible explanations might be responsible for the correlation.

It has been widely accepted that persistent chronic inflammation was prone to trigger cancerogenesis of normal cells [12]. Previous reports have demonstrated that inflammatory response, by promotion of angiogenesis and suppression of immune response, will create a hospitable microenvironment in which the survival, expansion and epigenetic changes of premalignant cells can be supported and promoted [38]. As described in the published literature, CRLM patients who were with presence of an inflammatory response carried a more aggressive tumor biological behavior and a higher rate of tumor recurrence [39].

Recent data have indicated that NLR denotes the balance between the inflammatory response and antitumor immune function. Neutrophils are regarded as the main source of vascular endothelial growth factor (VEGF), which acts as a proangiogenic mediator in tumor related angiogenesis, and therefore accelerates the progression of malignancy [40–42]. Meanwhile, a heightened amount of neutrophils levers the up-regulation of cytokines and chemokines (interleukin-1, interleukin-6 (IL-6), or tumor necrosis factors), thus facilitating tumor proliferation [12,16]. As reported, inflammatory activation of IL-6 were considered to aid the neoplastic and metastatic progression of CRLM [43] and that serum levels of IL-6 are found associated with stage and presence of metastases in CRC [44,45]. Comparatively, lymphocytes, as crucial elements in the immune system (innate immunity and the adaptive immune response), will induce cytotoxic cell death and cytokine secretion to eradicate tumor cells [46]. Ropponen KM reported that the elevation of tumor-infiltrating lymphocytes (TILs) independently predicted a good survival in colorectal cancer [47]; a decrease in lymphocyte count symbolized a weakened immune defense against tumors [48]. In addition, vitro assay found that increased neutrophils in peripheral blood inhibited the cytolytic activity of lymphocytes and natural killer cells to tumor cells [49]. Hence, heightened NLR, resulted from either an increase of neutrophils or a decrease of lymphocytes, marked the potential suppression of host immune surveillance and response to malignancy. Taken together, the features and mechanisms aforementioned explained the clinical finding that long-term survival inferiority was found in patients with high pretreatment NLR value. Our findings are in agreement with the results from Neal CP who found that elevated pretreatment NLR independently predicted poor prognosis in both univariate and multivariate analysis for patients with CRLM. Median OS length of high NLR patients (NLR≥5) was only 27.8 months when compared to 39.8 months for low NLR patients (NLR<5) [32]. Similarly, the report by Halazun KJ documented that patients with NLR≥5 carrying a five year RFS rate of 12% in comparison with 42% in patients with NLR<5 (p<0.0001) [27]. Subgroup estimations in this meta-analysis demonstrated that prognostic significances of increased pretreatment NLR for poor OS were identified according to five predefined parameters. Of note, although similar results of subgroup analyses for RFS were obtained by most of predefined parameters, negative predictive role of heightened NLR was found in the subgroup of NLR cutoff value = 2.5 (HR 1.30, 95% CI 0.91–1.85). Such inconsistent result might be biased, due to the limited studies for inclusion (only two studies). Furthermore, NLR carried advantages of cost-effectiveness, general availability and easy reproducibility over other serum markers. As was reflected in the present meta-analysis, an increased pretreatment NLR was strongly linked with worse long-term survival in patients with CRLM. Therefore, NLR can be routinely monitored as a marker for survival prediction in CRLM patients, regardless of therapeutic intervention (surgical resection or non-surgical treatment).

Our present study has two main strengths. (1) To date, to our best knowledge, this is the first meta-analysis addressing the association between NLR and survival outcomes as well as clinicopathological features in CRLM patients. (2) Heterogeneities for OS and RFS in our meta-analysis were not evident (OS: I^2^ 0, RFS: I^2^ 45.20%), and coherent results were accordingly produced by both subgroup and sensitivity analyses. Hence, our results were reliable and robust.

In spite of the above-mentioned strengths, certain limitations of the present study should be taken into consideration. The main limitation was that the size of the studies included is rather small, despite of the fact that a comprehensive and extensive search strategy was implemented in the major medical databases (PubMed, EMBASE, the Cochrane Library, Web of Science and the Chinese SinoMed). Besides, most of the enrolled studies were retrospective, which relatively influenced the accuracy of the results. Furthermore, differences in cutoff value definition (cutoff value = 5 in six studies and cutoff value = 2.5 in two studies) probably led to heterogeneity and variability, which might preclude the application of these ratios and our results in clinical practice. In addition, in one of included studies, HR and corresponding 95% CI for RFS was retrieved from Kaplan-Meier curves because of the unavailability of these values from the article and the absence of reply from the authors [27]. The consistency and accuracy of the results might thus be affected. Finally, because of the small size of the studies for inclusion in subgroup analysis, the statistical power of results might be potentially weakened; likewise, the result that no significant correlations between NLR values and clinicopathological features of CRLM were identified should be interpreted with much caution due to only two or three studies available for inclusion. Nevertheless, the current study undoubtedly represented one more step in developing a persuasive argument for the prognostic significance of NLR for CRLM.

## Conclusion

In summary, the results of this systematic review and meta-analysis suggested that an elevated pretreatment NLR value was closely correlated with poor long-term survival (OS and RFS) in CRLM patients. Hence, NLR can serve as a useful and cost-effective marker with strong prognostic significance in CRLM patients received either surgical resection or non-surgical treatment. Further multicenter prospective studies are required to back up the conclusion and identify the definitive cutoff value of NLR.

## Supporting Information

S1 PRISMA Checklist PRISMA checklist. (DOC)

S1 Table Characteristics of included studies. (DOC)

## Abbreviations

NLR: neutrophil-lymphocyte ratio
NOS score: Newcastle-Ottawa Scale score
CRC: Colorectal cancer
CRLM: colorectal liver metastasis
OS: overall survival
RFS: recurrence free survival
RFA: radiofrequency ablation
