# Supplementary material for: Prognostic Significance of Neutrophil-to-Lymphocyte Ratio in Colorectal Liver Metastasis: A Systematic Review and Meta-Analysis
Source: PLoS One. 2016 Jul 18;11(7):e0159447. doi: 10.1371/journal.pone.0159447 (PMC4948783; doi:10.1371/journal.pone.0159447)
Supplement: S1 Table — (DOC) [file pone.0159447.s002.doc]

**Supporting Information**

**S1 PRISMA Checklist.** PRISMA checklist.

**S1 Table Characteristics of included studies.**

| **First author** | **Year** | **Type** | **Region** | **Period** | **Patients characteristics** | | | | **Treatment**  (n) | **NLR** | | **End**  **point** | **Maximum**  **follow-up**  (months) | **NOS**  **score** |
| --- | --- | --- | --- | --- | --- | --- | --- | --- | --- | --- | --- | --- | --- | --- |
|  |  |  |  |  | **Total** | Age | Male (%) | High NLR (n) |  | Cutoff | Sample  time, site |  |  |  |
| Neal C.P(2015)^32^ | 2015 | R | The UK | 2006-2010 | 302 | ≥65 (n=168) | 63.58 | 53 | SR | 5 | PT, PB | OS | 29.7(4-96)† | 7 |
| Giakoustidis A^26^ | 2015 | P | The UK | 2005-2012 | 169 | >70 (n=34) | 61.54 | 71 | SR | 2.5 | PT, PB | OS,  RFS | 34.6† | 8 |
| Chang ZH^33,#^ | 2014 | R | China | 2000-2010 | 98 | 62(28–92)† | 57.14 | 45 | pRFA | 2.5 | PT, PB | RFS | 35.2±21.8¶ | 6 |
| Zeman M^34^ | 2013 | R | Poland | 2001-2009 | 96 | NA | NA | 17 | SR | 5 | PT, PB | RFS | 44(6-156)¶ | 6 |
| Zhang Y^35^ | 2012 | R | China | 2000-2008 | 92 | 59(43-78)† | NA | 21 | pRFA | 5 | PT, PB | OS,  RFS | 27.1±9.8¶ | 8 |
| Neal C.P(2011)^36^ | 2011 | R | The UK | 2000-2006 | 198 | ≥65 (n=73) | 61.62 | 28 | SR | 5 | PT, PB | OS,  RFS | 40(6-70)† | 6 |
| Kishi Y(SR)^37^ | 2009 | R | The USA | 1997-2007 | 200 | 57(23-86)† | 66.00 | 20 | SR | 5 | PT, PB | OS | 28(2-102)† | 6 |
| Kishi Y(Non SR)^37^ | 2009 | R | The USA | 1997-2007 | 90 | 56(26-81)† | 67.78 | 14 | Chemo | 5 | PT, PB | OS | 16(3-99)† | 6 |
| Halazun KJ^27^ | 2008 | R | The UK | 1996-2006 | 440 | 64±10.7 | 65.68 | 78 | SR | 5 | PT, PB | OS,  RFS | 24(11-97)† | 8 |

**S Table 1 Characteristics of included studies**

NLR: neutrophil-lymphocyte ratio; NOS: Newcastle-Ottawa Scale; R: retrospective; P: prospective; SR: surgical resection; pRFA: percutaneous radiofrequency ablation; Chemo: chemotherapy; PT: pretreatment; PB: peripheral blood; OS: overall survival; RFS: recurrence free survival; NA: not available. ¶: value is mean with range in parenthesis or with standard deviation; †: value is median with range in parenthesis; ^#^ : studies from the database of the Chinese SinoMed.
