# Supplementary material for: Expression of Concern: Prognostic Significance of Neutrophil-to-Lymphocyte Ratio in Colorectal Liver Metastasis: A Systematic Review and Meta-Analysis
Source: PLoS One. 2023 Jul 3;18(7):e0288268. doi: 10.1371/journal.pone.0288268 (PMC10317213; doi:10.1371/journal.pone.0288268)
Supplement: S1 File — (ZIP) [file pone.0288268.s001.zip › CEA.pdf]

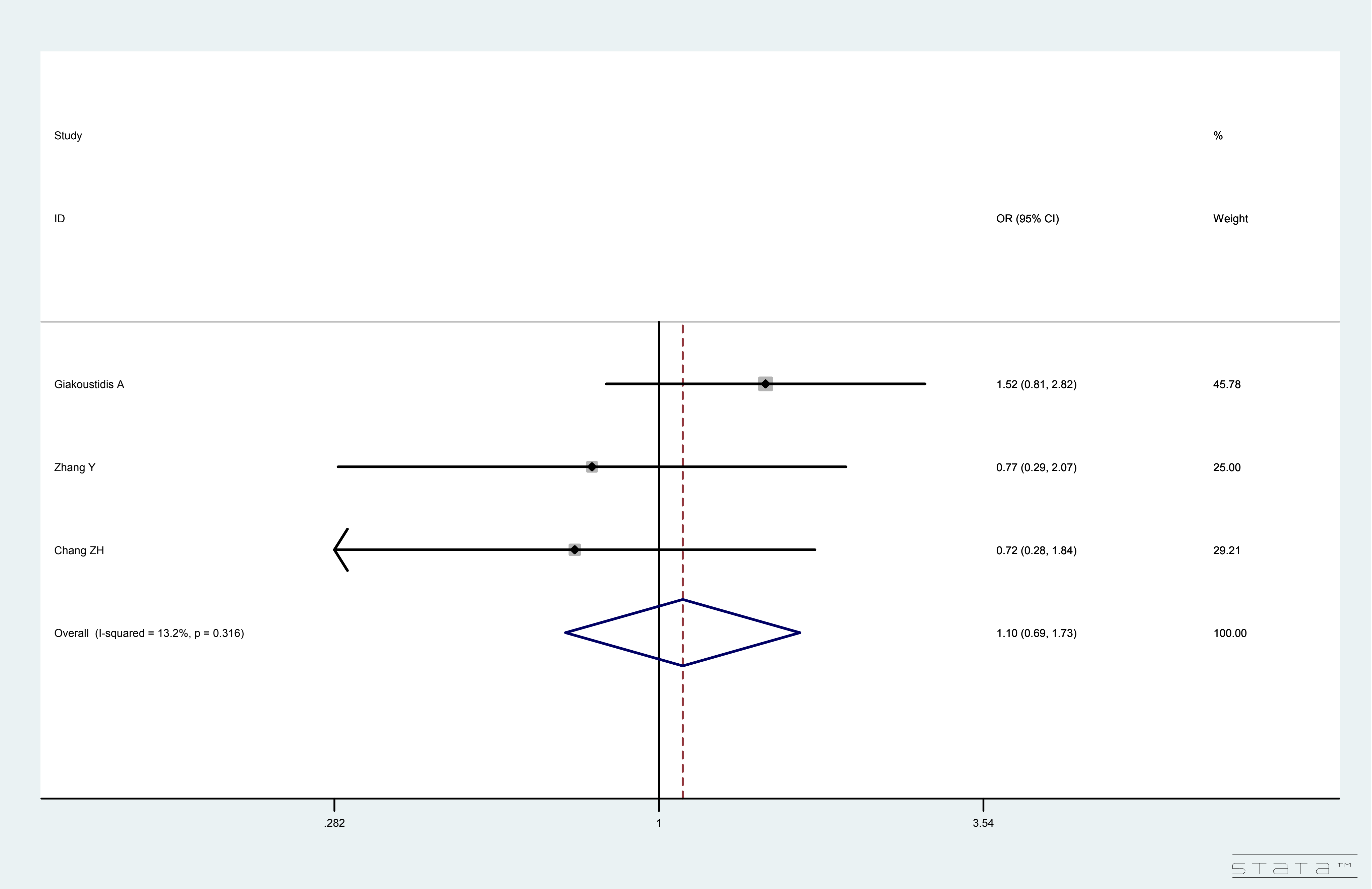
Study
%
ID
OR (95% CI)
Weight
Giakoustidis A
1.52 (0.81, 2.82)
45.78
Zhang Y
0.77 (0.29, 2.07)
25.00
Chang ZH
0.72 (0.28, 1.84)
29.21
Overall (I-squared = 13.2%, p = 0.316)
1.10 (0.69, 1.73)
100.00
.282
1
3.54
