# Supplementary material for: Expression of Concern: Prognostic Significance of Neutrophil-to-Lymphocyte Ratio in Colorectal Liver Metastasis: A Systematic Review and Meta-Analysis
Source: PLoS One. 2023 Jul 3;18(7):e0288268. doi: 10.1371/journal.pone.0288268 (PMC10317213; doi:10.1371/journal.pone.0288268)
Supplement: S1 File — (ZIP) [file pone.0288268.s001.zip › no. metastases.pdf]

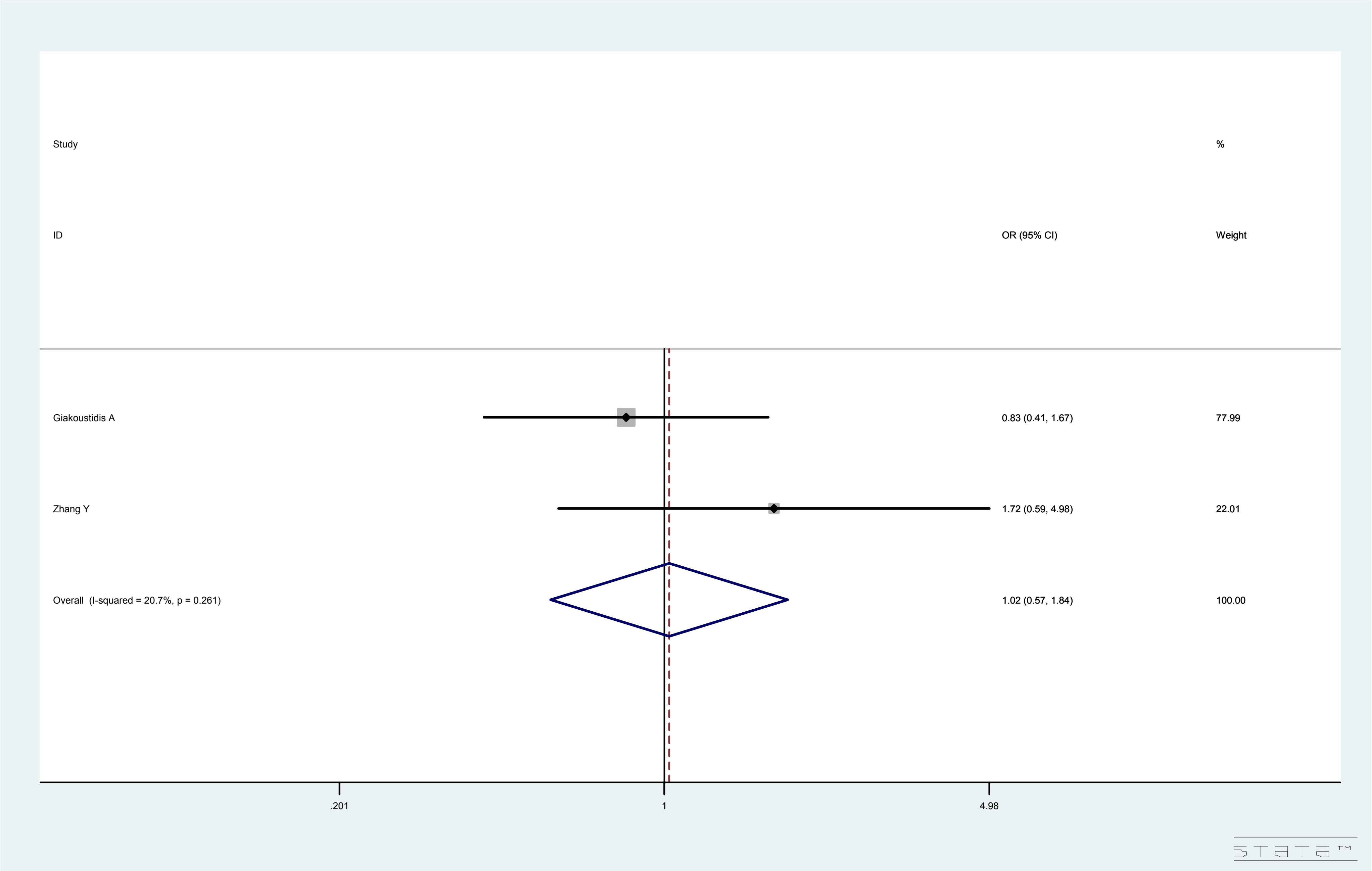
Study
%
ID
OR (95% CI)
Weight
Giakoustidis A
0.83 (0.41, 1.67)
77.99
Zhang Y
1.72 (0.59, 4.98)
22.01
Overall (I-squared = 20.7%, p = 0.261)
1.02 (0.57, 1.84)
100.00
.201
1
4.98
