# Supplementary figures and images for: Expression of Concern: Prognostic Significance of Neutrophil-to-Lymphocyte Ratio in Colorectal Liver Metastasis: A Systematic Review and Meta-Analysis
Source: PLoS One. 2023 Jul 3;18(7):e0288268. doi: 10.1371/journal.pone.0288268 (PMC10317213; doi:10.1371/journal.pone.0288268)

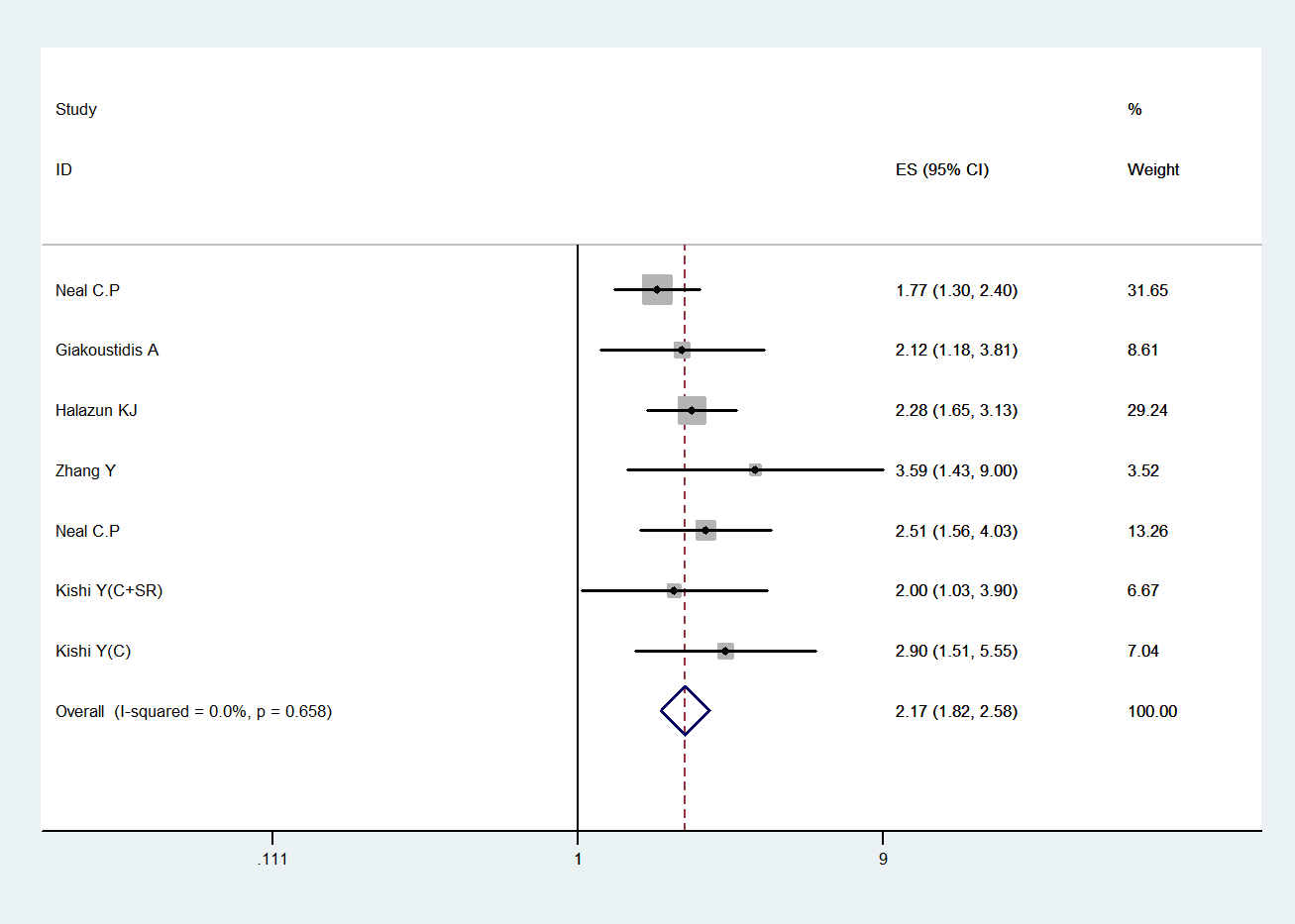

Supplement: S1 File — (ZIP) [file pone.0288268.s001.zip › 6分为亚组,fixed OS.tif]

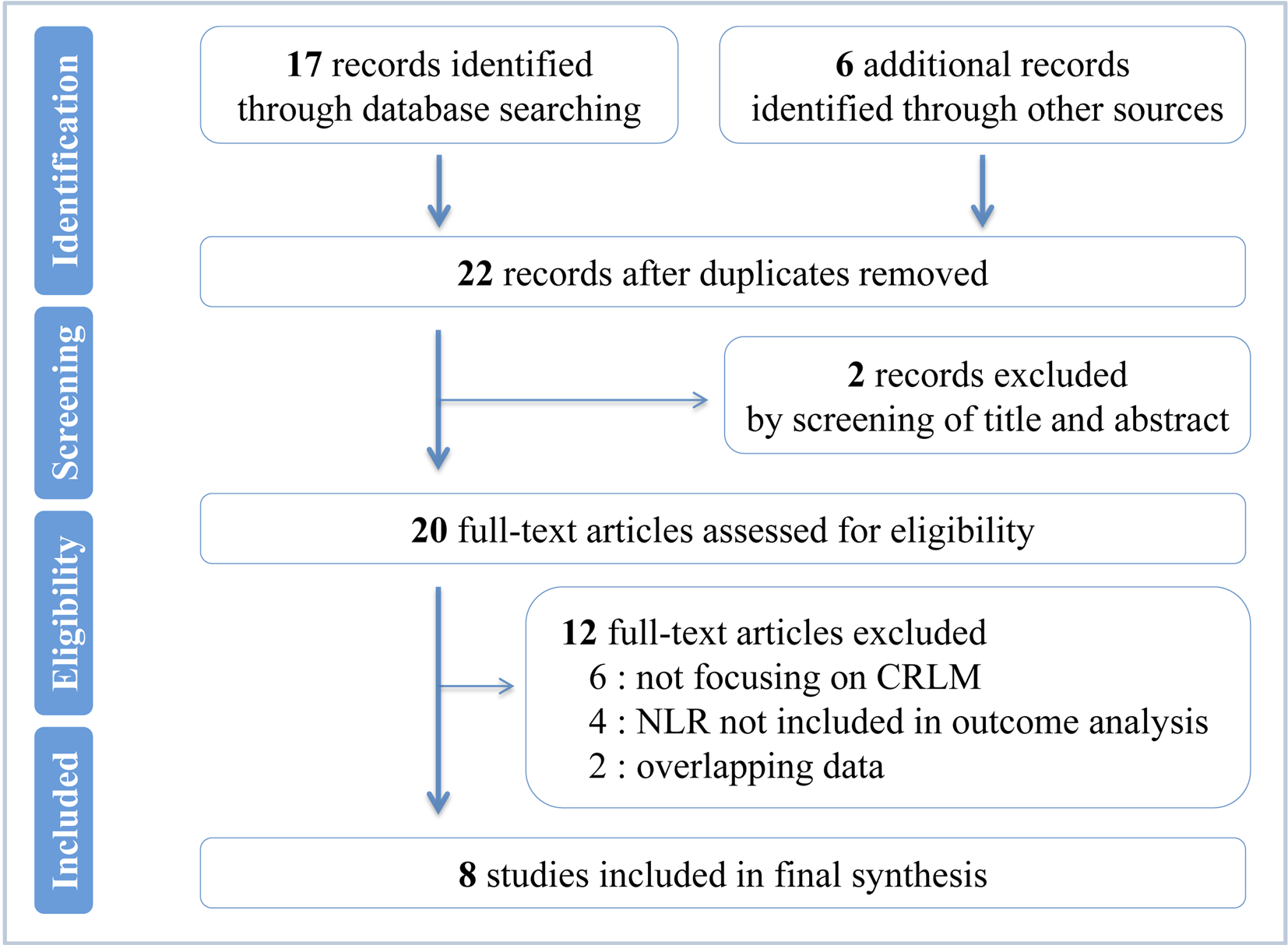

Supplement: S1 File — (ZIP) [file pone.0288268.s001.zip › Fig 1.tif]

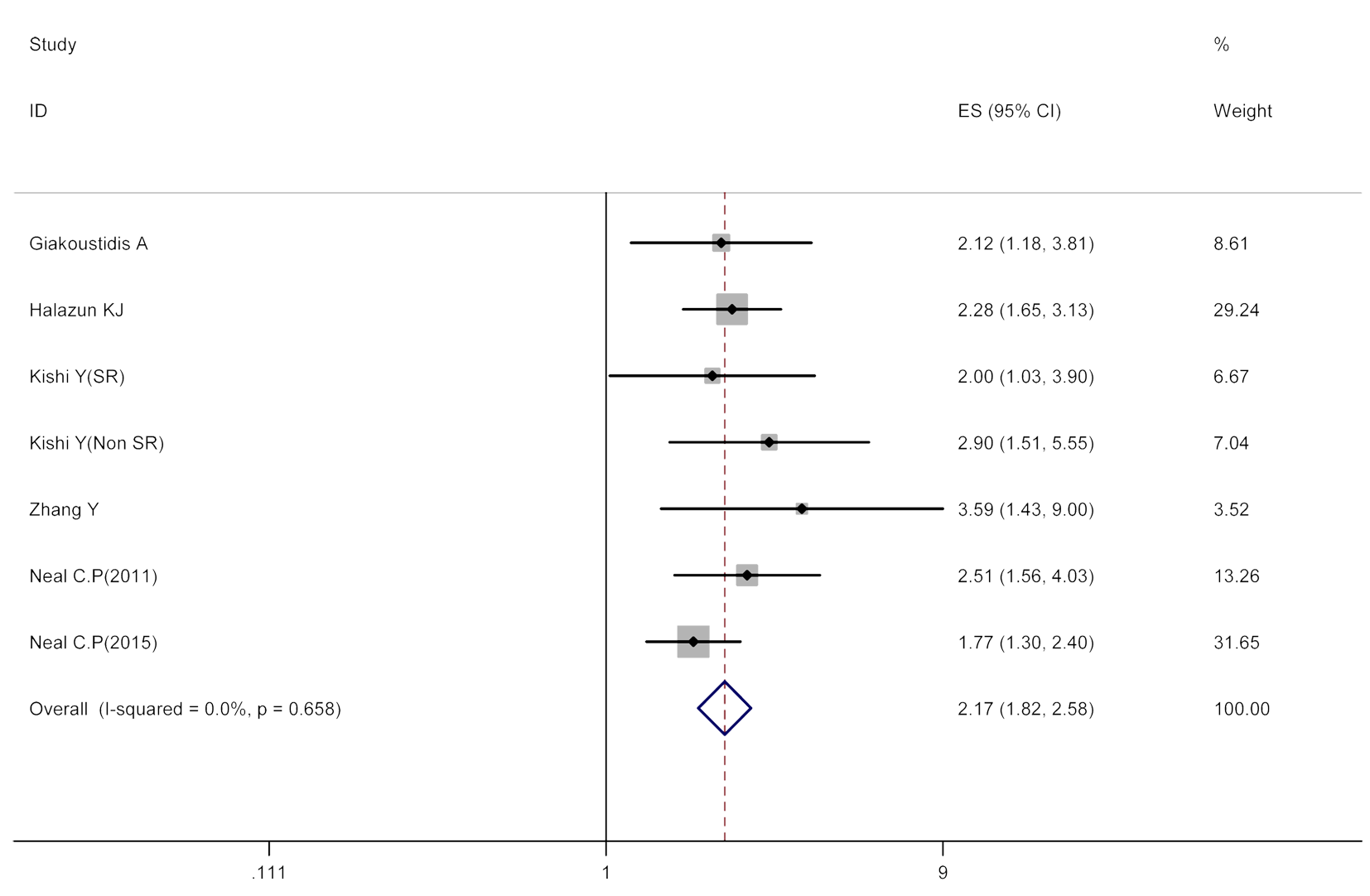

Supplement: S1 File — (ZIP) [file pone.0288268.s001.zip › Fig 2.tif]

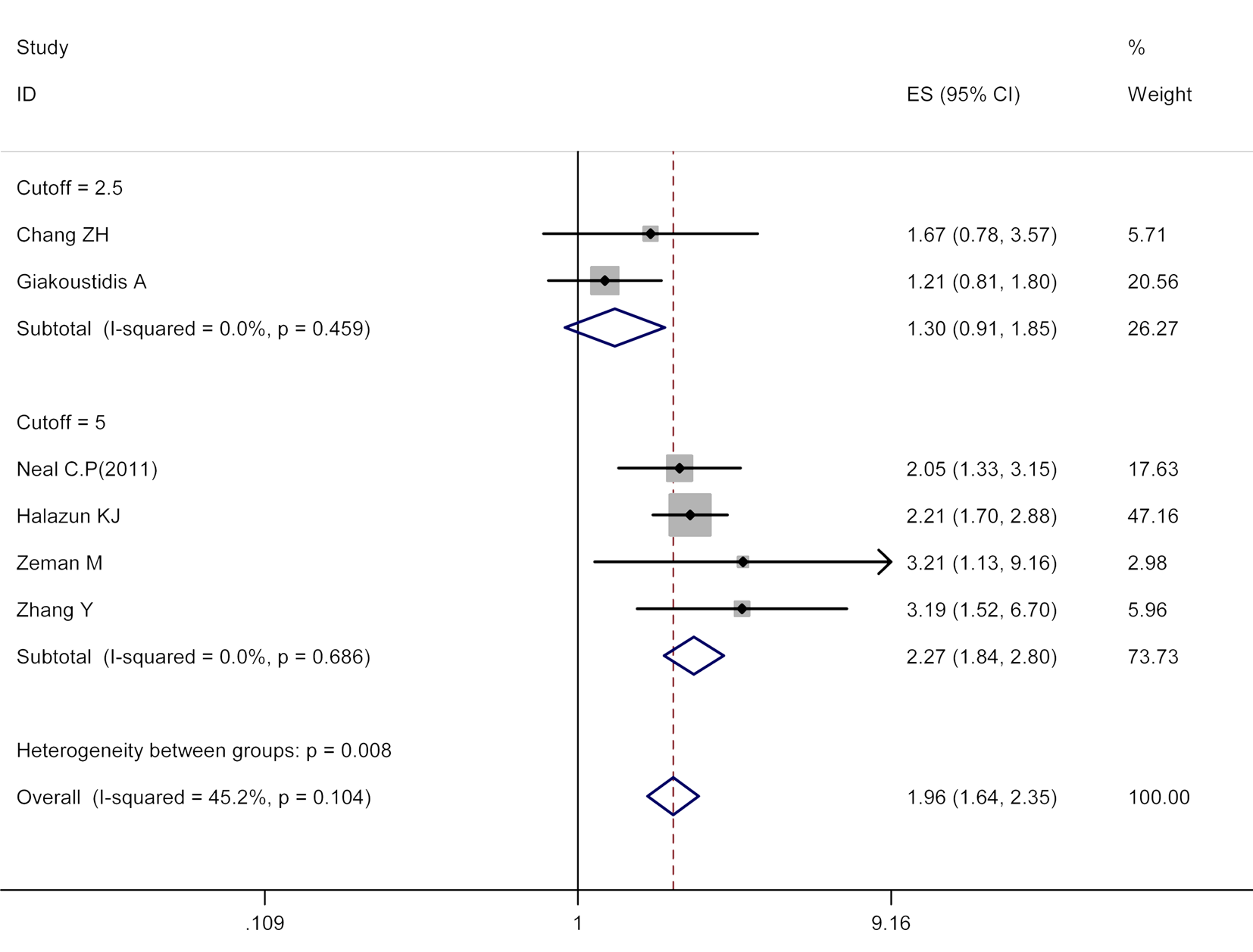

Supplement: S1 File — (ZIP) [file pone.0288268.s001.zip › Fig 3.tif]

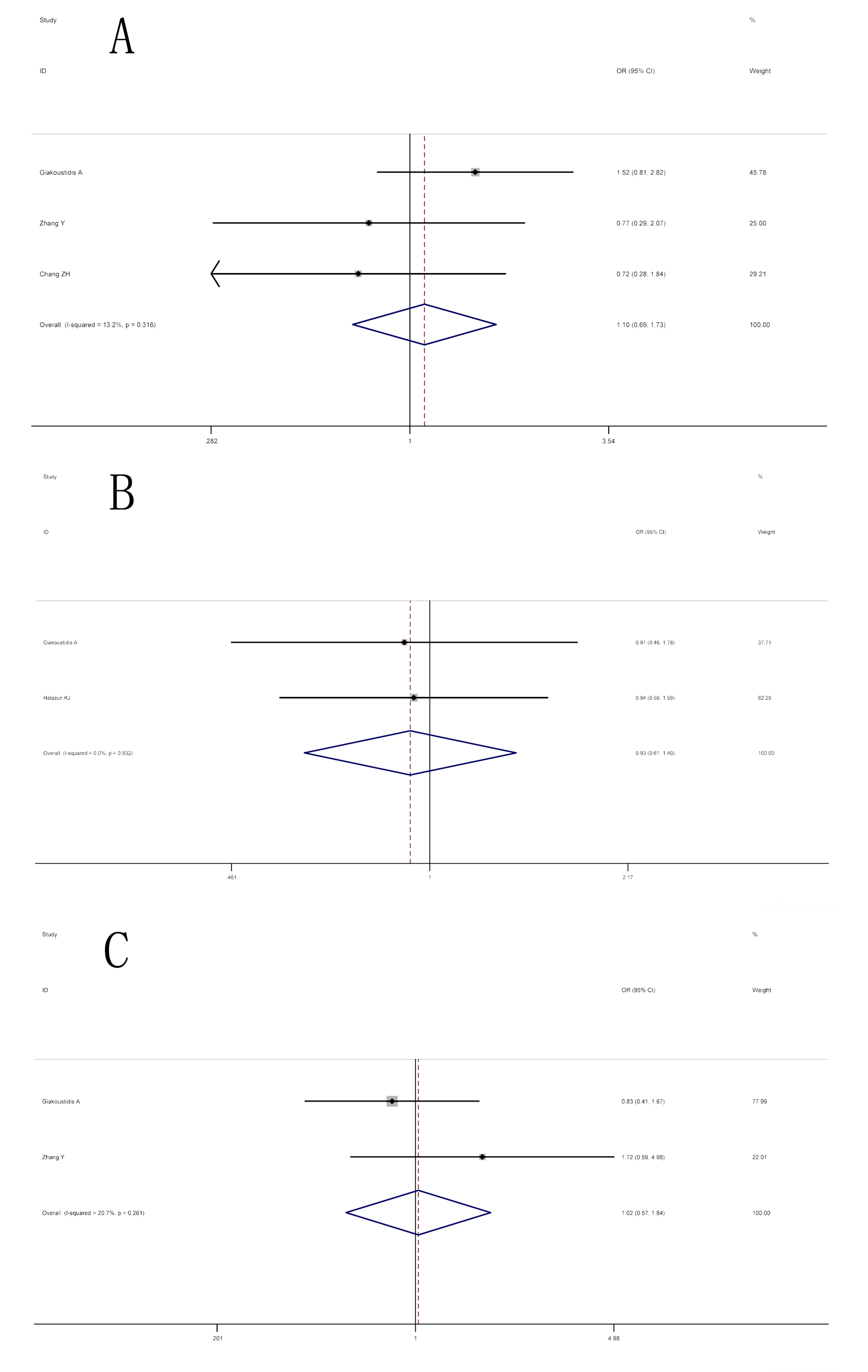

Supplement: S1 File — (ZIP) [file pone.0288268.s001.zip › Fig 4.tif]

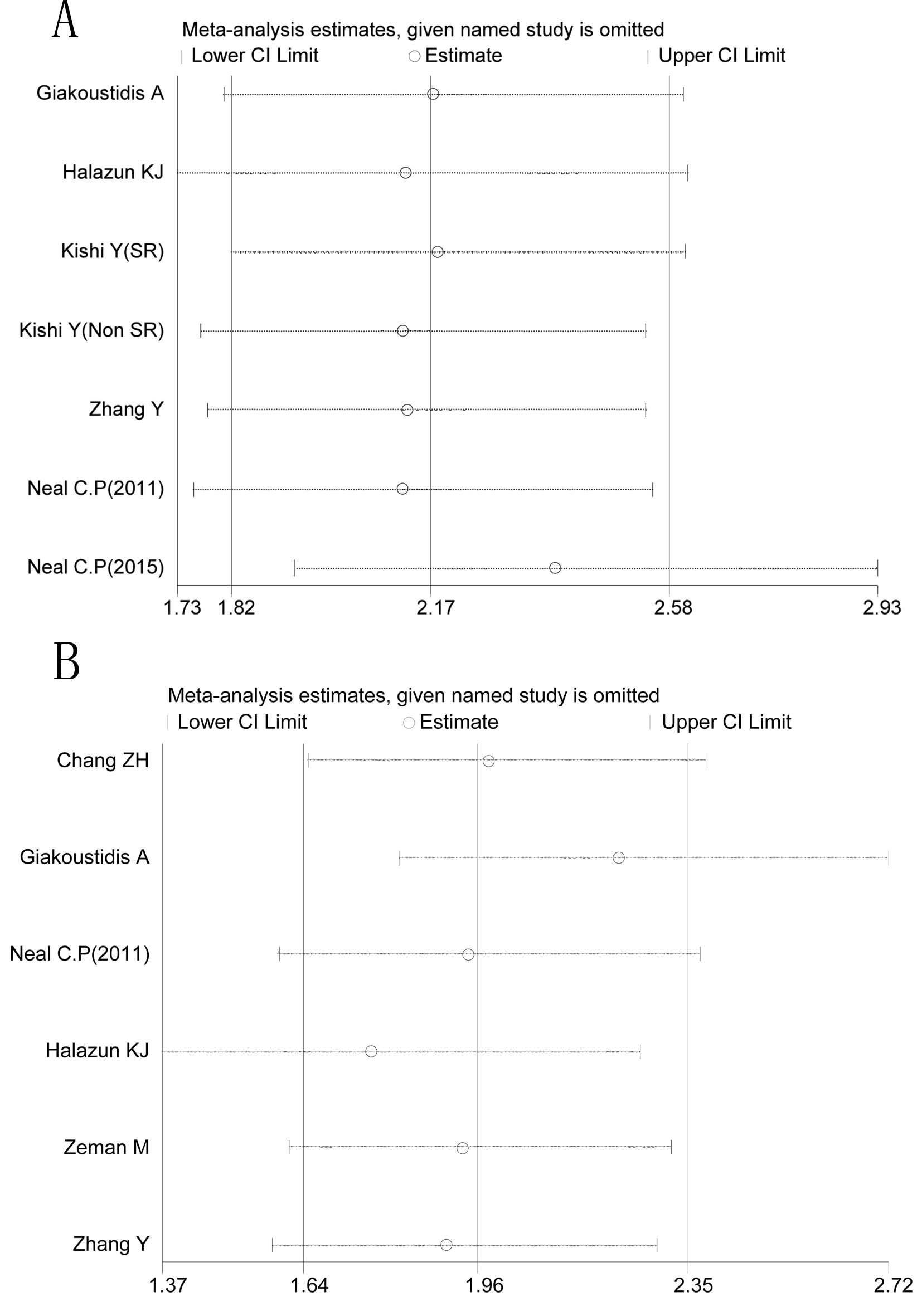

Supplement: S1 File — (ZIP) [file pone.0288268.s001.zip › Fig 5.tif]

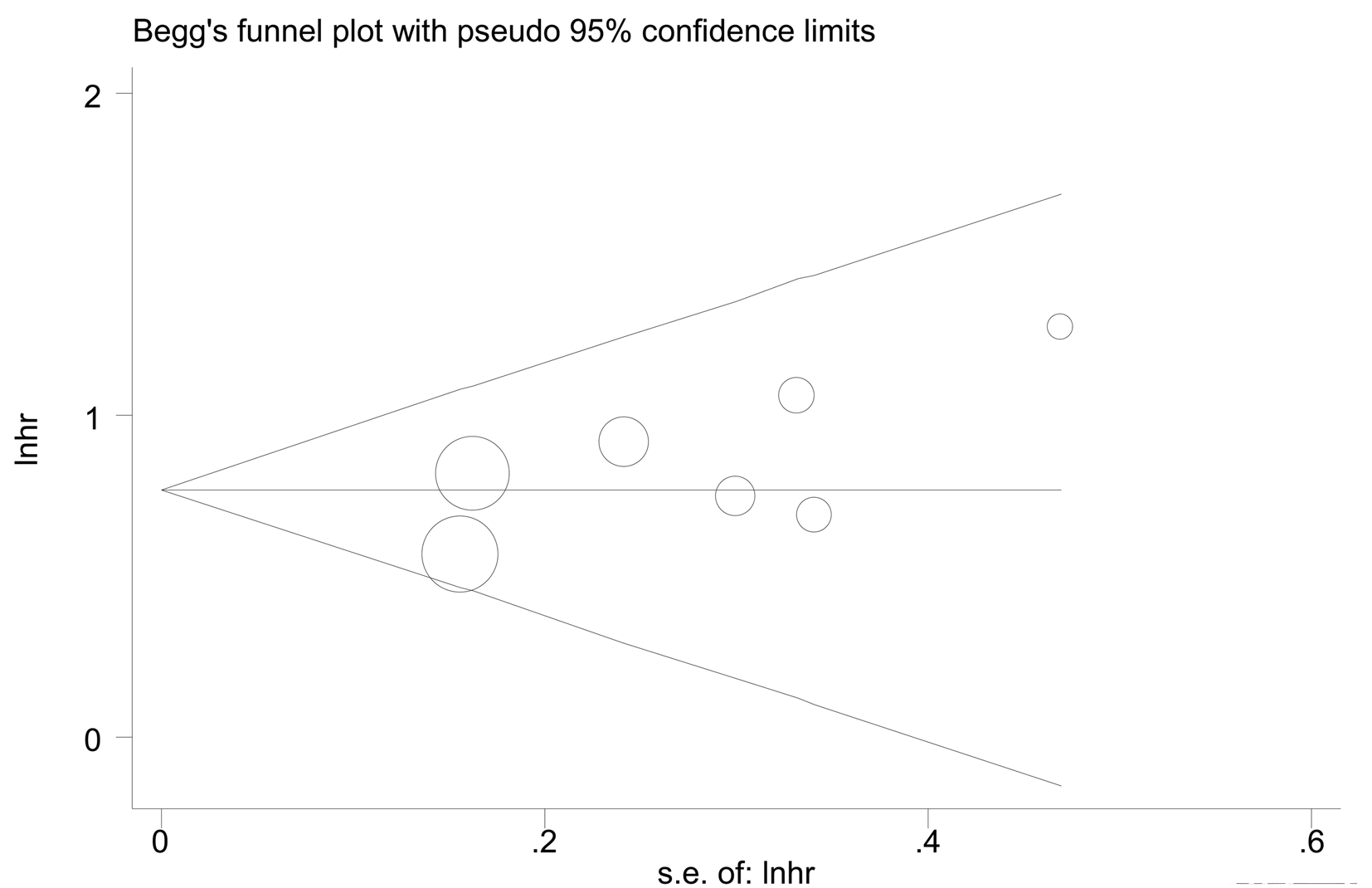

Supplement: S1 File — (ZIP) [file pone.0288268.s001.zip › Fig 6.tif]

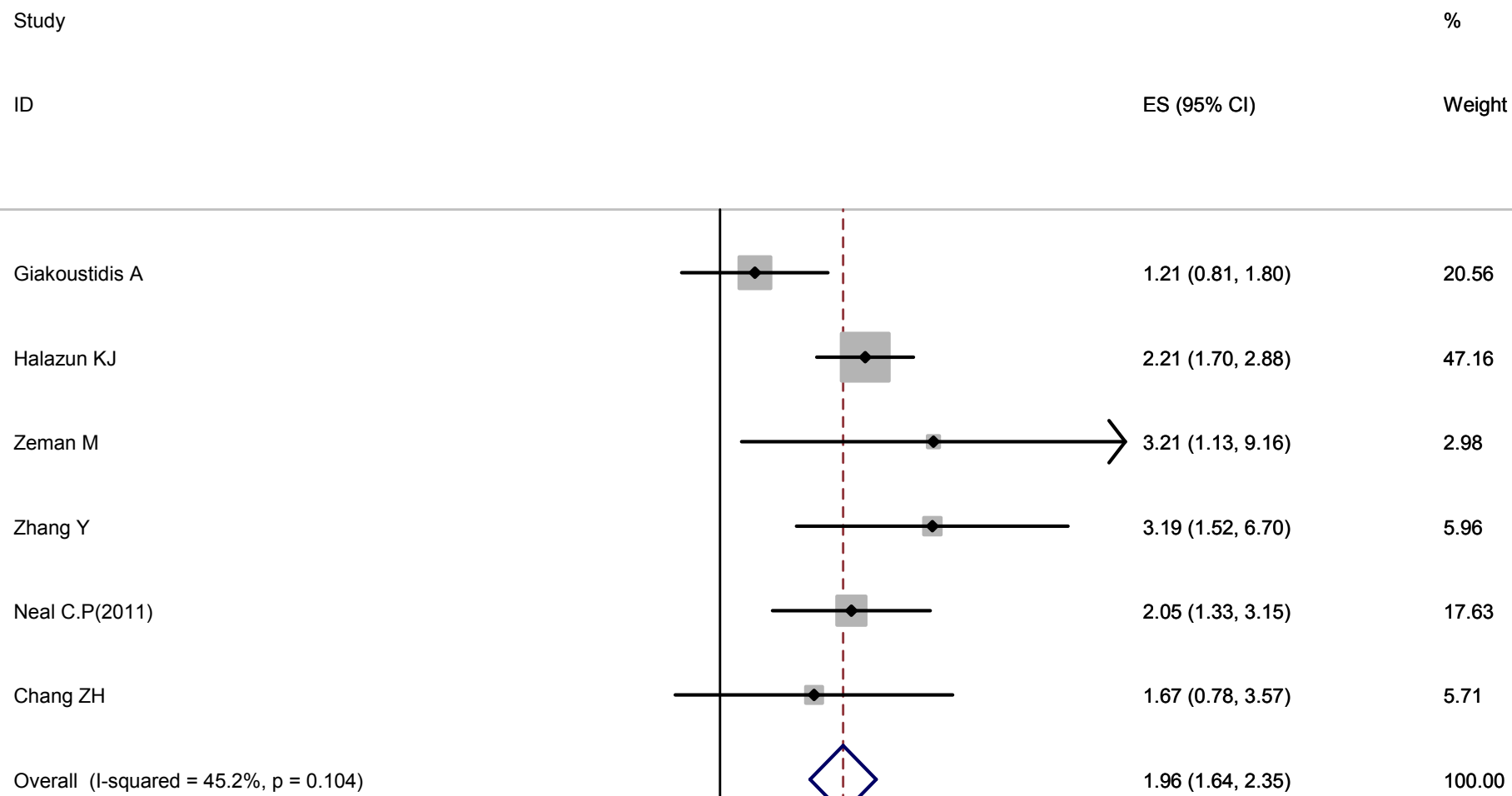

.109

1

9.16

Supplement: S1 File — (ZIP) [file pone.0288268.s001.zip › fixed DFS.pdf]

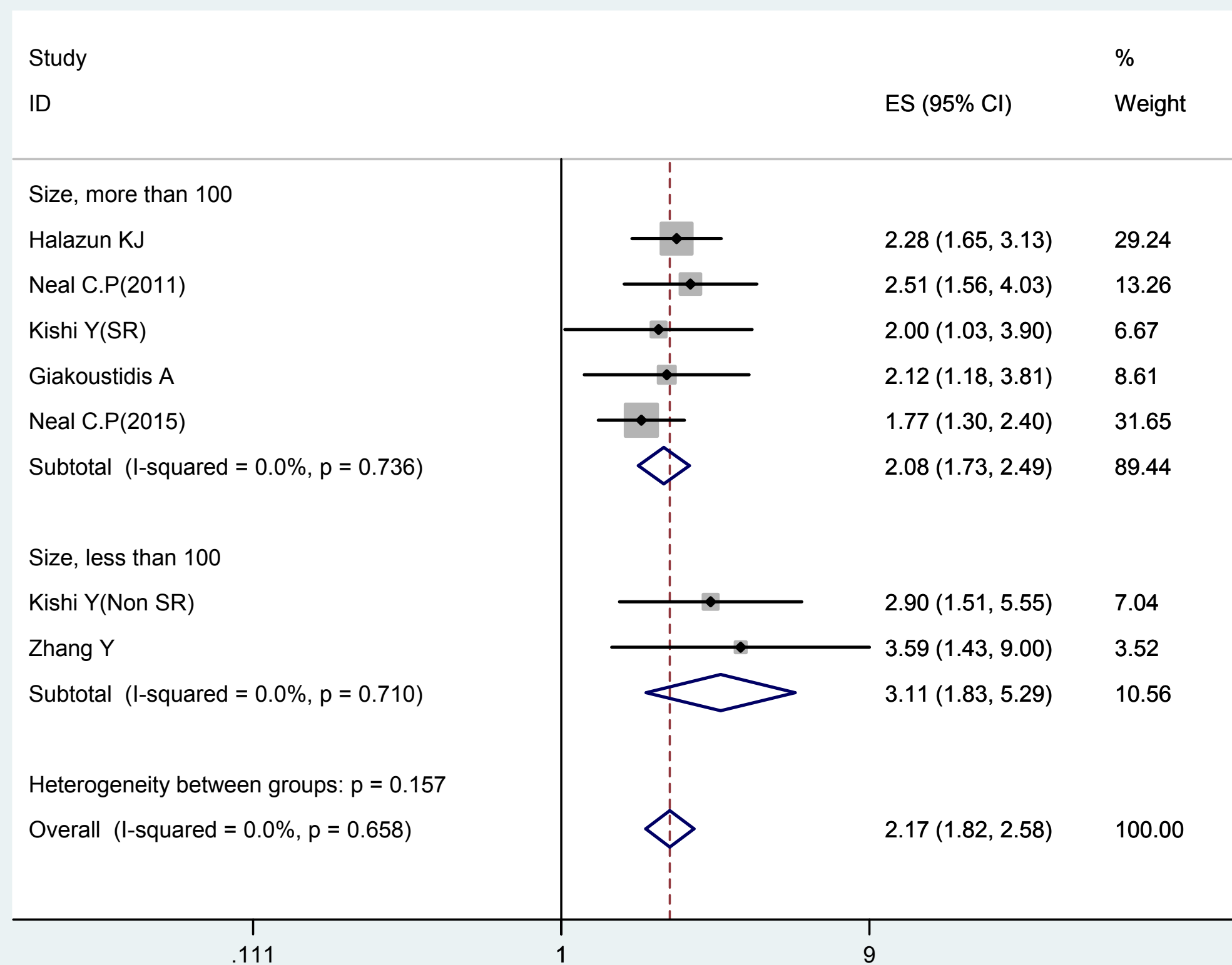

Supplement: S1 File — (ZIP) [file pone.0288268.s001.zip › fixed OS 带亚组 cutoff.pdf]

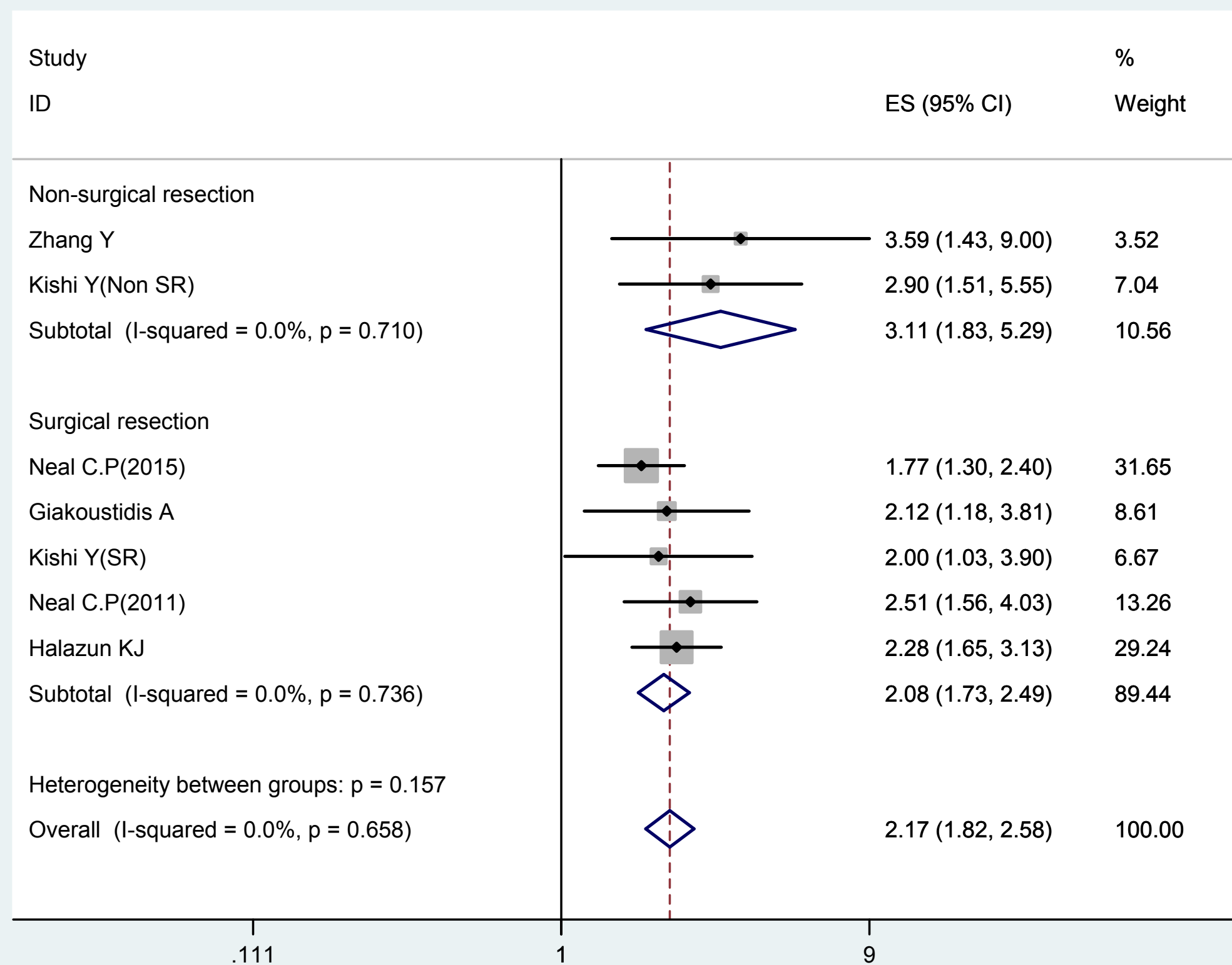

Supplement: S1 File — (ZIP) [file pone.0288268.s001.zip › fixed OS 带亚组 treatment.pdf]

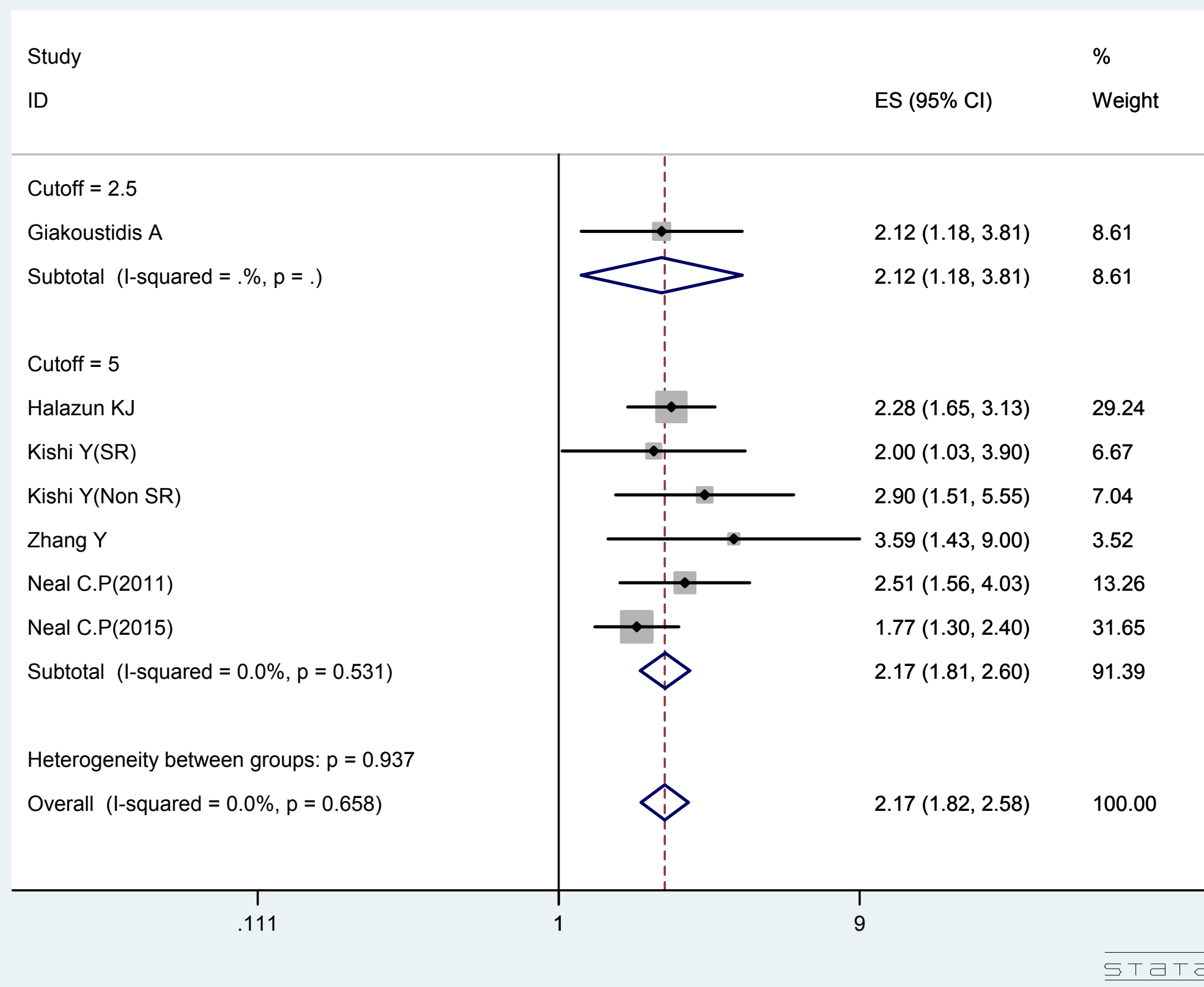

Supplement: S1 File — (ZIP) [file pone.0288268.s001.zip › fix、.pdf]

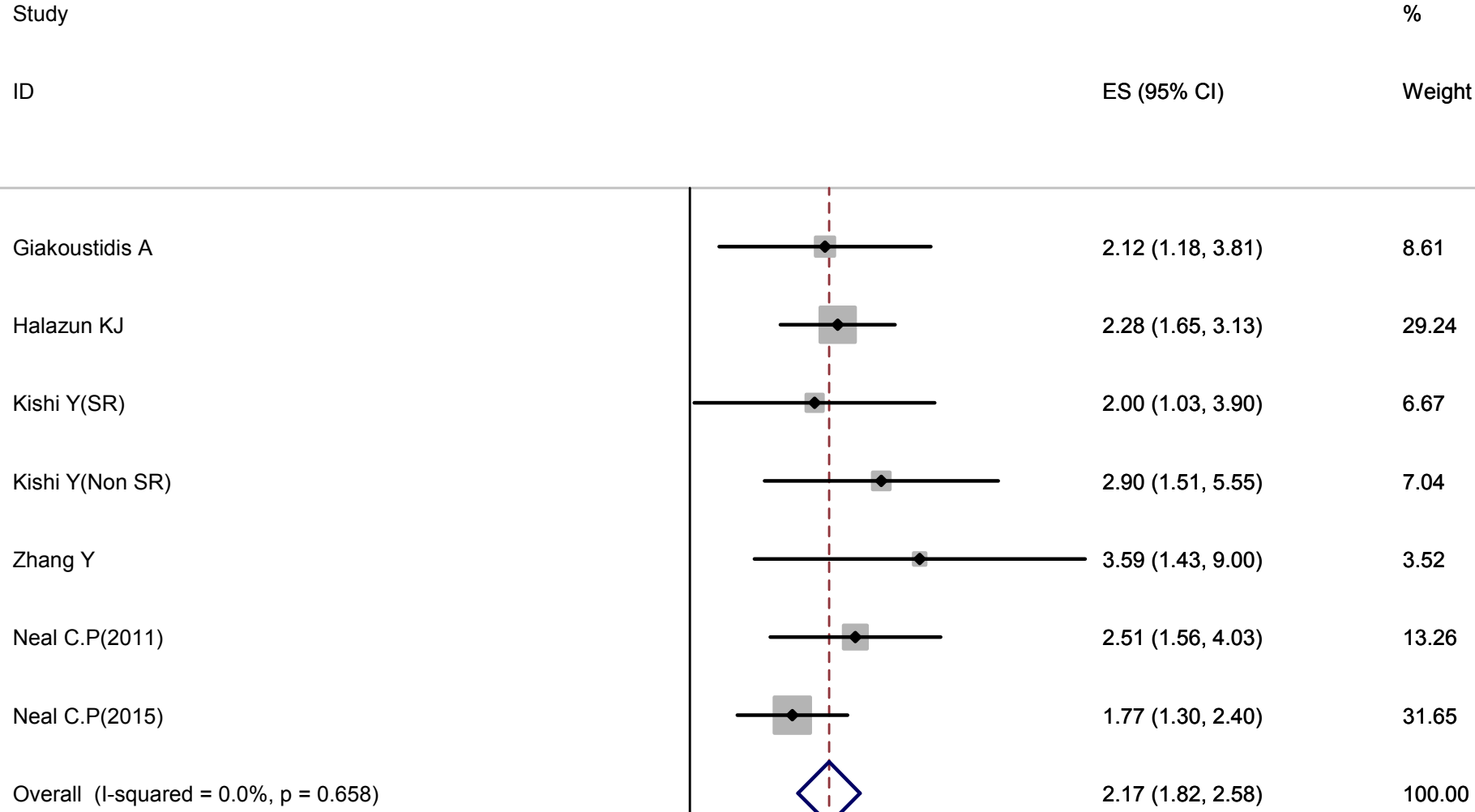

.111

1

9

Supplement: S1 File — (ZIP) [file pone.0288268.s001.zip › OS 总体 不分亚组.pdf]

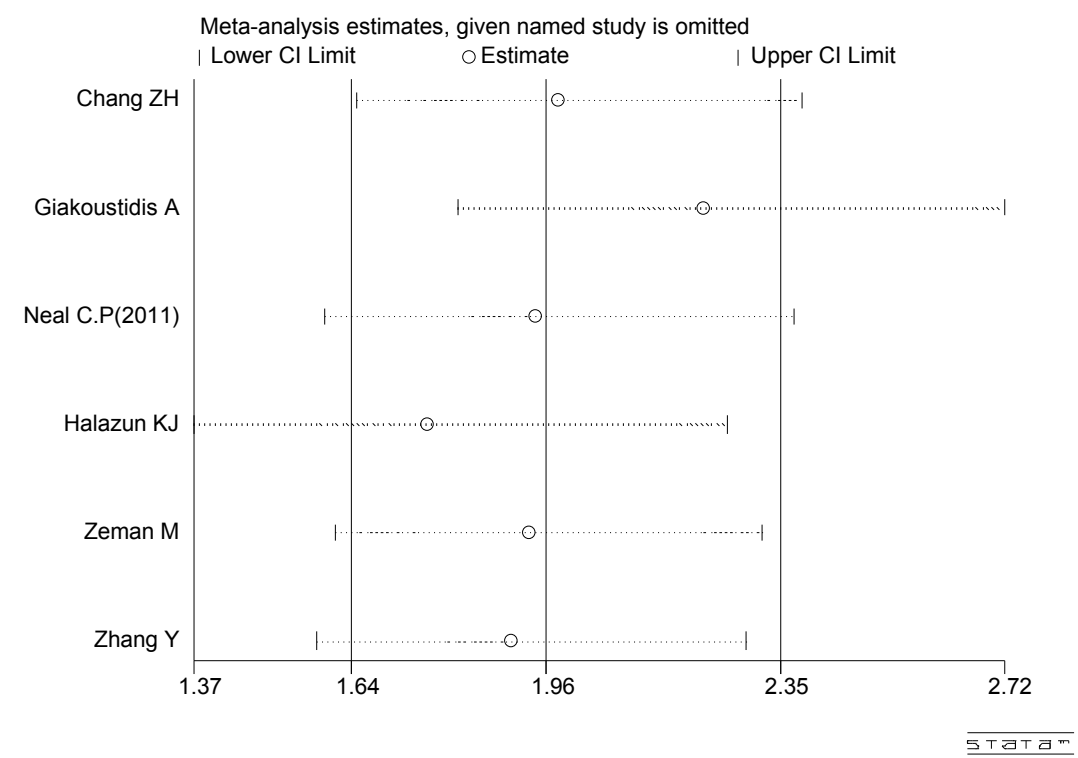

Supplement: S1 File — (ZIP) [file pone.0288268.s001.zip › RFS - 复件.pdf]

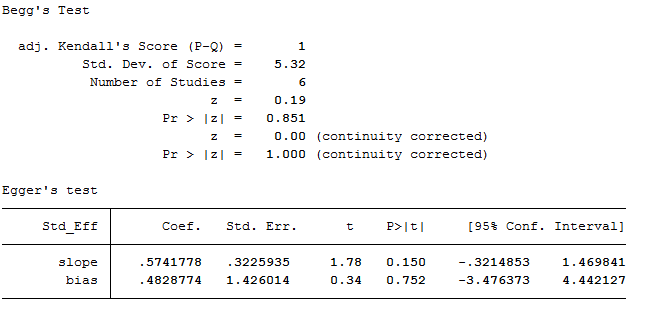

Supplement: S1 File — (ZIP) [file pone.0288268.s001.zip › RFS 发表偏倚 截图.png]

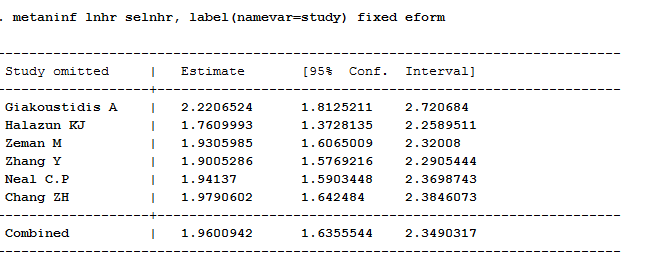

Supplement: S1 File — (ZIP) [file pone.0288268.s001.zip › RFS 敏感性分析 截图.png]

Begg's funnel plot with pseudo 95% confidence limits

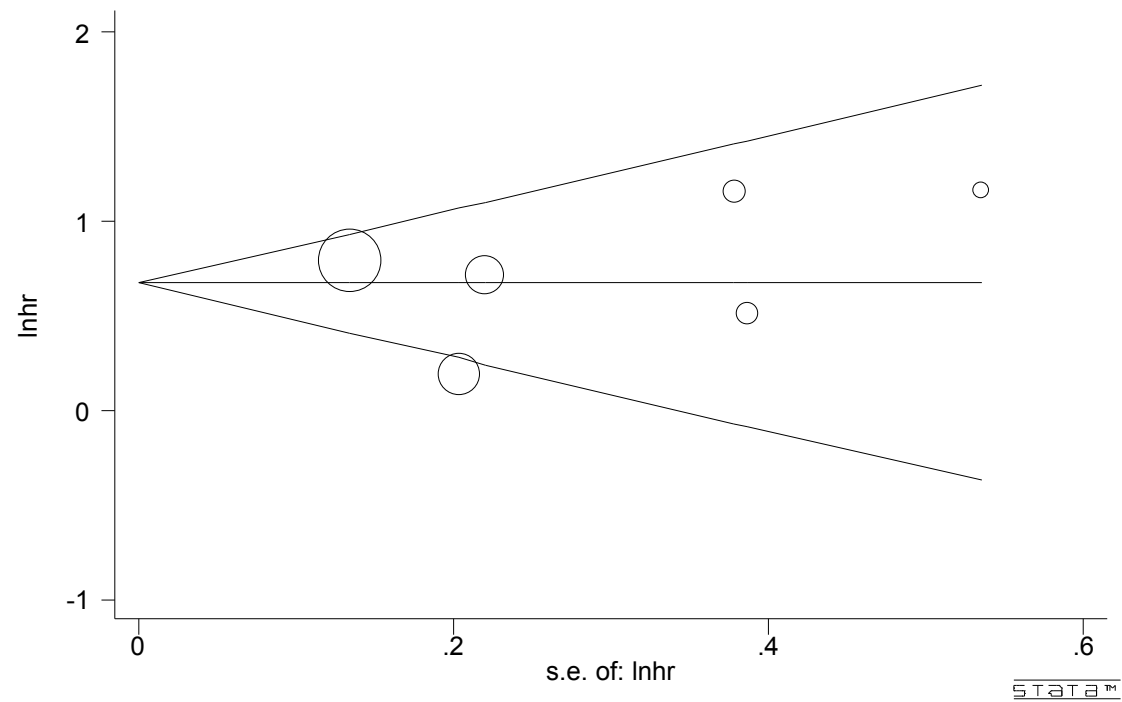

Supplement: S1 File — (ZIP) [file pone.0288268.s001.zip › RFS.pdf]

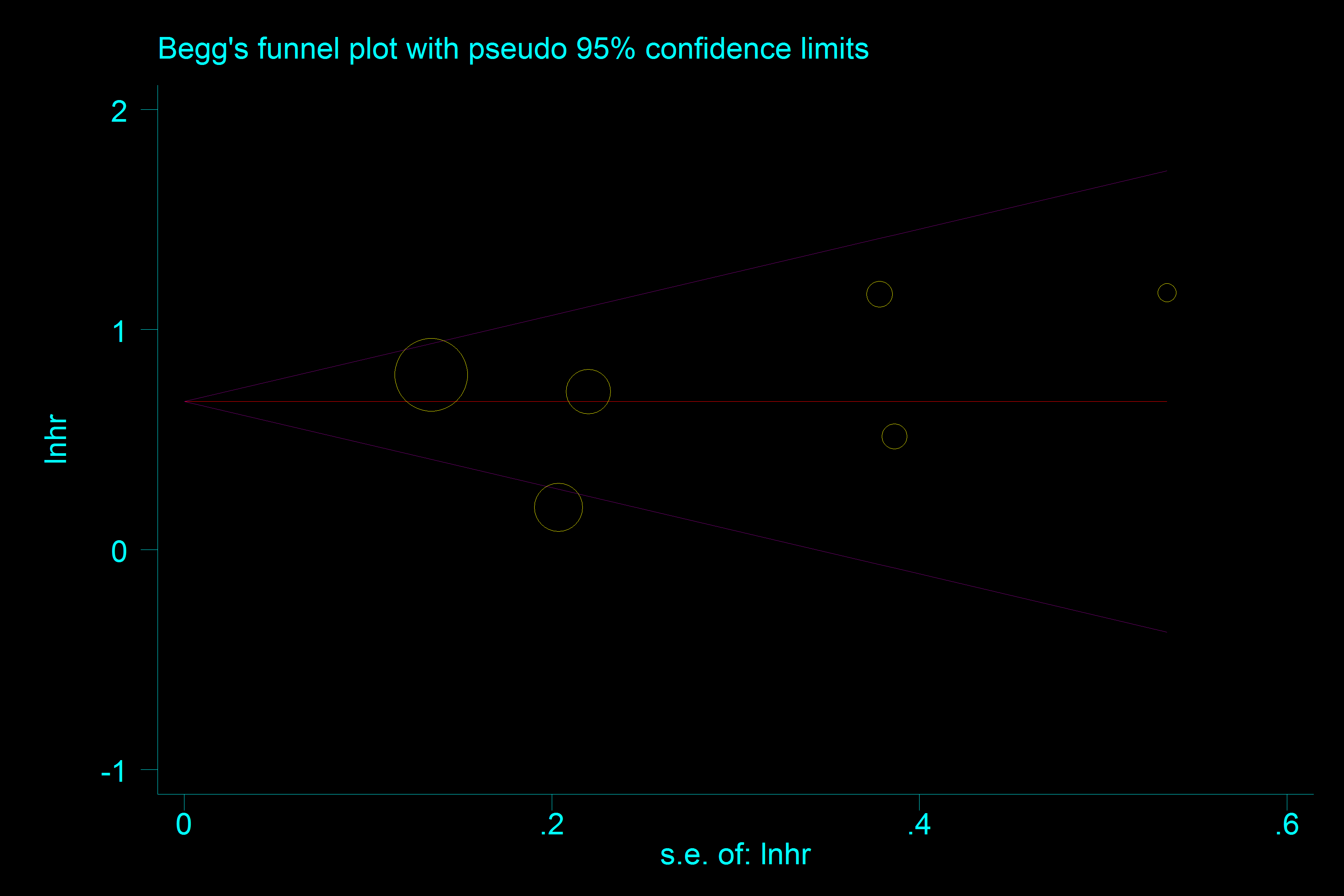

Supplement: S1 File — (ZIP) [file pone.0288268.s001.zip › RFS.tif]

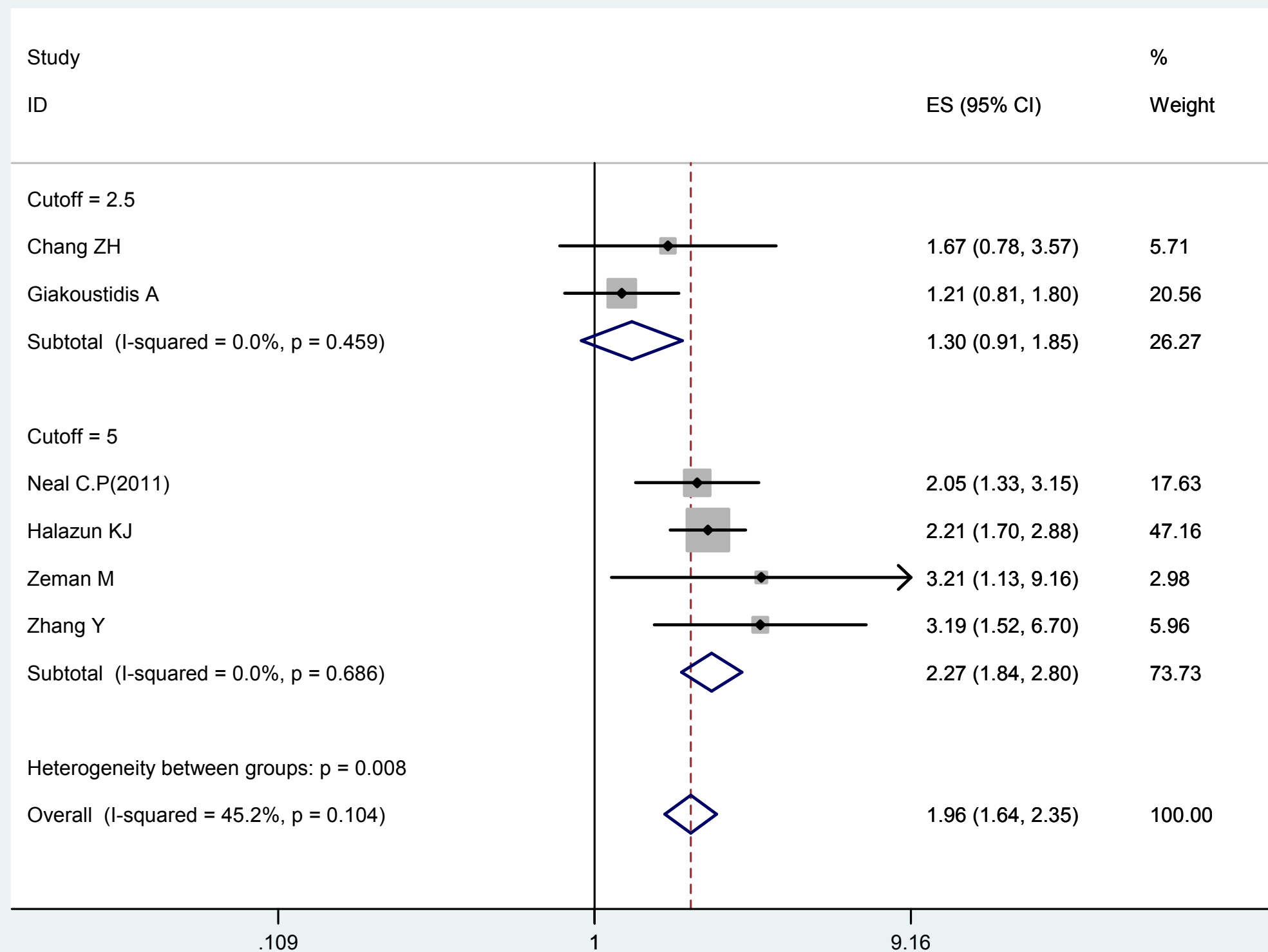

Supplement: S1 File — (ZIP) [file pone.0288268.s001.zip › Stata graph Fixed RFS 带亚组cutoff.pdf]

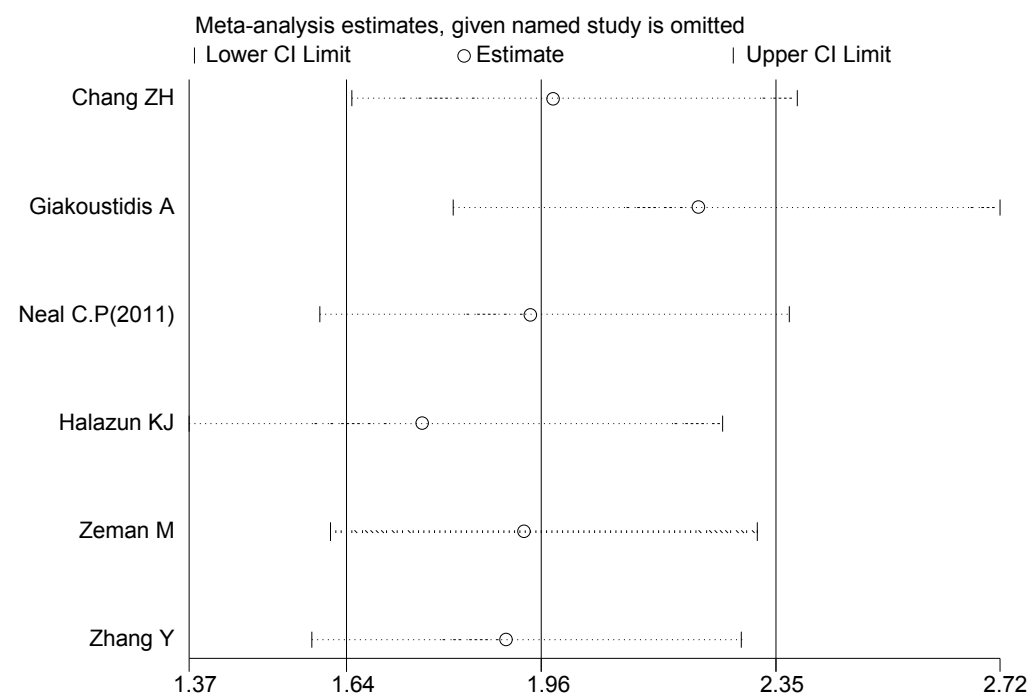

Supplement: S1 File — (ZIP) [file pone.0288268.s001.zip › Stata graph.pdf]

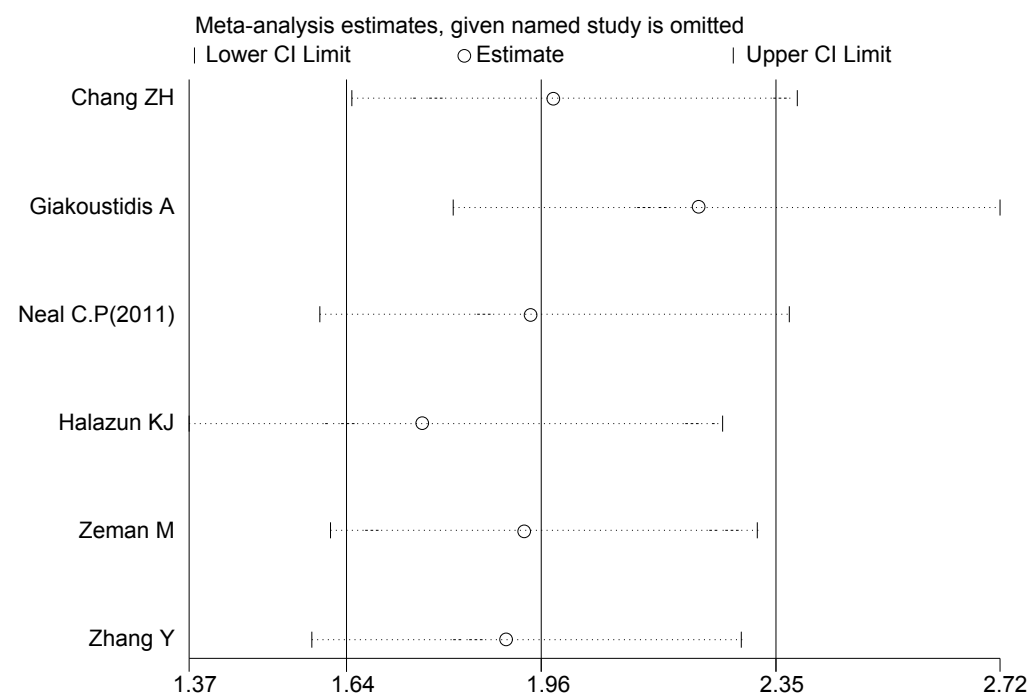

Supplement: S1 File — (ZIP) [file pone.0288268.s001.zip › Stata graph1200.pdf]

Study

%

ID

OR (95% CI)

Weight

Giakoustidis A

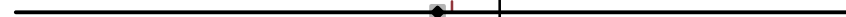

0.91 (0.46, 1.78)

37.71

Halazun KJ

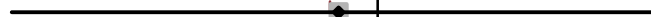

0.94 (0.56, 1.59)

62.29

Overall (I-squared = 0.0%, p = 0.932)

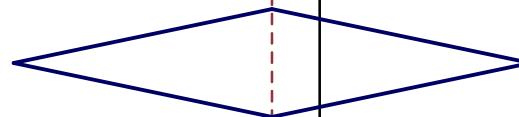

0.93 (0.61, 1.40)

100.00

.461

1

2.17

Supplement: S1 File — (ZIP) [file pone.0288268.s001.zip › time of.pdf]

Begg's funnel plot with pseudo 95% confidence limits

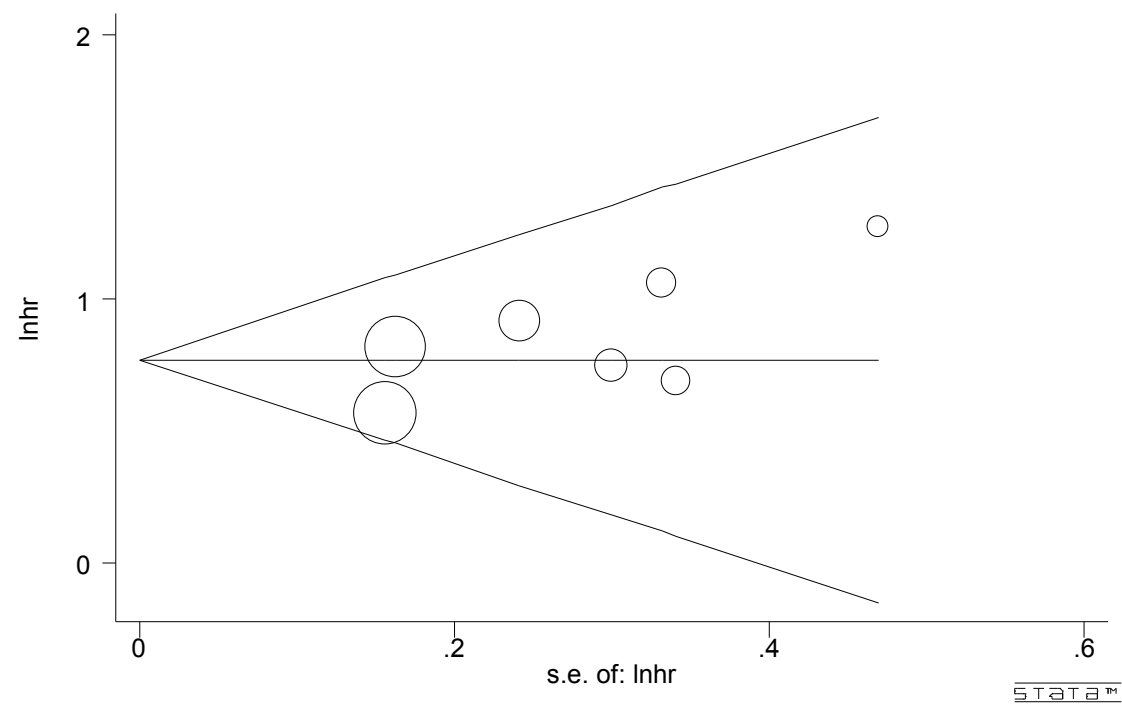

Supplement: S1 File — (ZIP) [file pone.0288268.s001.zip › 发表偏倚 OS.pdf]

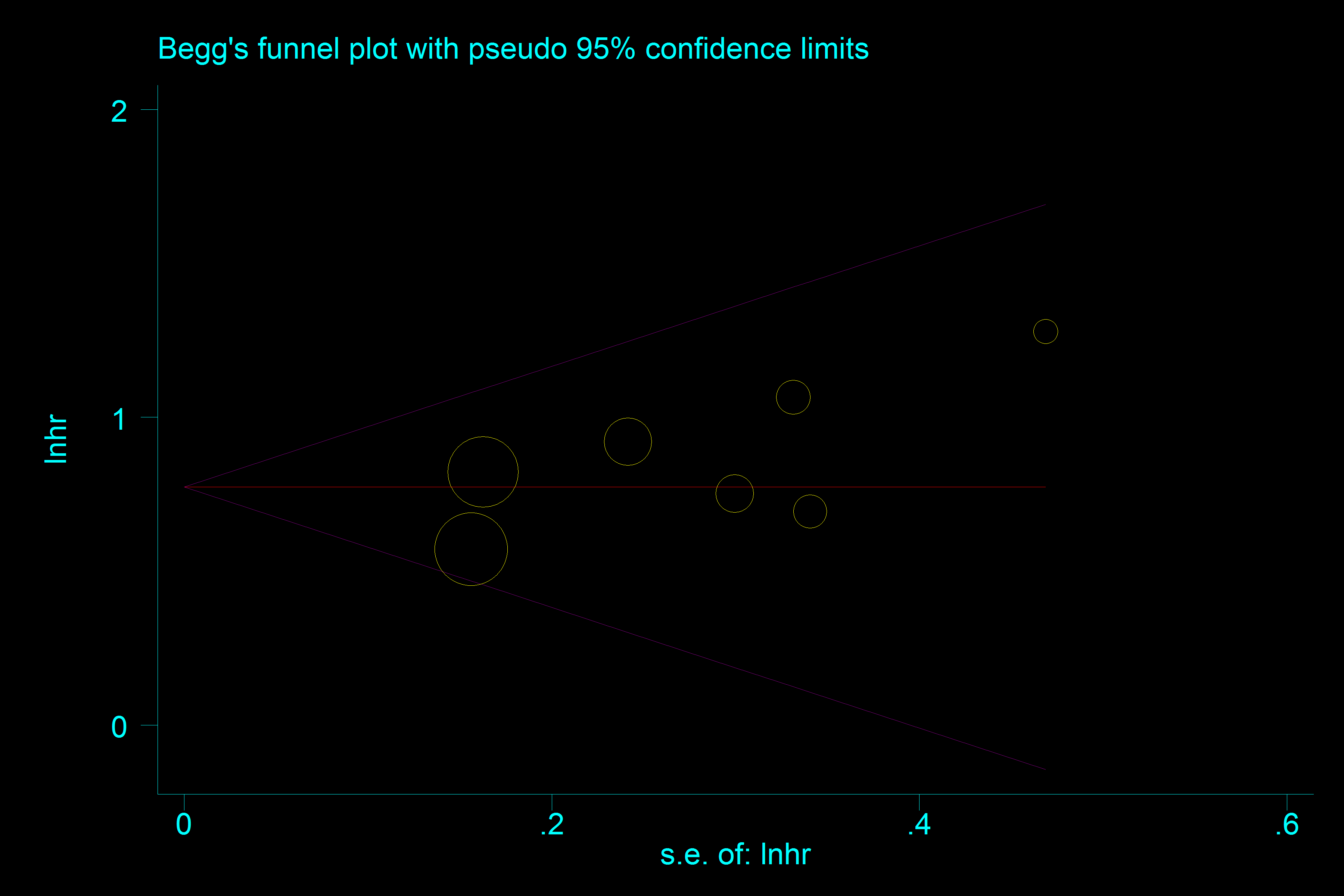

Supplement: S1 File — (ZIP) [file pone.0288268.s001.zip › 发表偏倚 OS.tif]

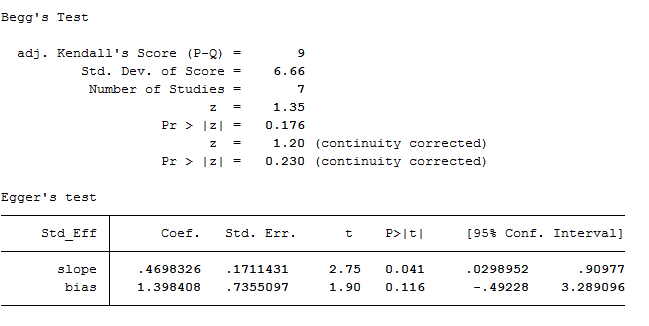

Supplement: S1 File — (ZIP) [file pone.0288268.s001.zip › 发表偏倚 截图.png]

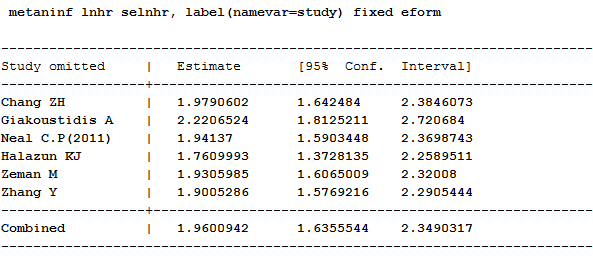

Supplement: S1 File — (ZIP) [file pone.0288268.s001.zip › 改名版.png]

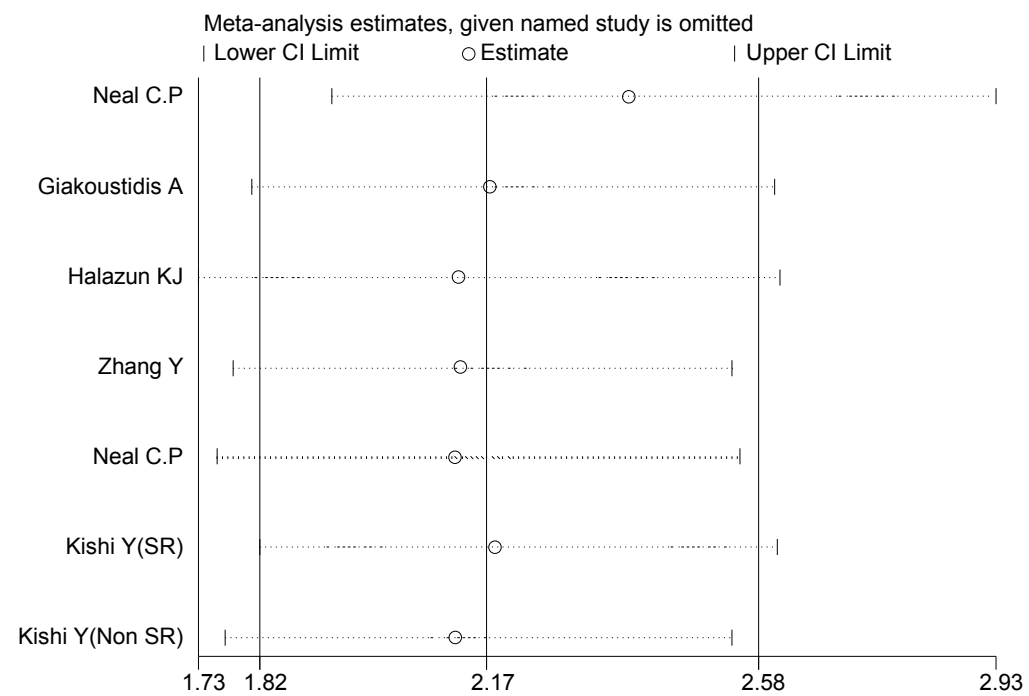

Supplement: S1 File — (ZIP) [file pone.0288268.s001.zip › 敏感性分析 OS.pdf]

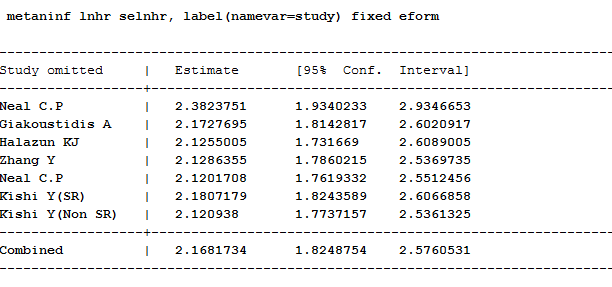

Supplement: S1 File — (ZIP) [file pone.0288268.s001.zip › 敏感性分析 截图.png]

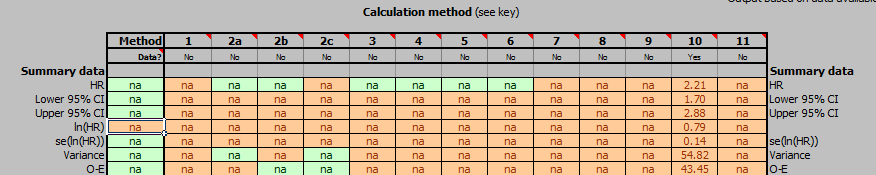

Supplement: S1 File — (ZIP) [file pone.0288268.s001.zip › 文章7 提取表格结果截图.png]

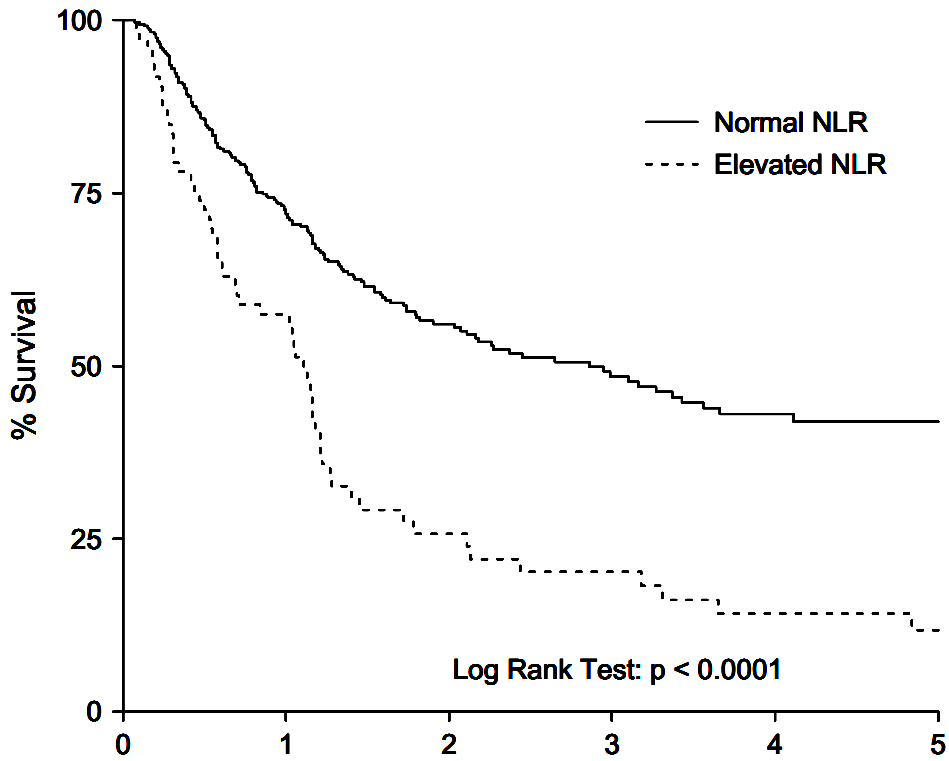

Supplement: S1 File — (ZIP) [file pone.0288268.s001.zip › 文章7 DFS HR提取.png]
